# The *Sinorhizobium meliloti* nitrogen-fixing symbiosis requires CbrA-dependent regulation of a DivL and CckA phosphorelay

**DOI:** 10.1128/jb.00399-23

**Published:** 2024-09-24

**Authors:** Hayden A. Bender, Roger Huynh, Charles Puerner, Jennifer Pelaez, Craig Sadowski, Elijah N. Kissman, Julia Barbano, Karla B. Schallies, Katherine E. Gibson

**Affiliations:** 1 Department of Biology, University of Massachusetts Boston, Boston, Massachusetts, USA; University of Massachusetts Chan Medical School, Worcester, Massachusetts, USA

**Keywords:** *Sinorhizobium meliloti*, cell cycle, symbiosis, CbrA, CtrA, CckA, DivL, alphaproteobacteria, *Caulobacter crescentus*, nitrogen fixation, *Medicago sativa*, bacteroid, Cell signaling

## Abstract

The cell cycle is a fundamental process involved in bacterial reproduction and cellular differentiation. For *Sinorhizobium meliloti*, cell cycle outcomes depend on its growth environment. This bacterium shows a tight coupling of DNA replication initiation with cell division during free-living growth. In contrast, it undergoes a novel program of endoreduplication and terminal differentiation during symbiosis within its host. While several DivK regulators at the top of its CtrA pathway have been shown to play an important role in this differentiation process, there is a lack of resolution regarding the downstream molecular activities required and whether they could be unique to the symbiosis cell cycle. The DivK kinase CbrA is a negative regulator of CtrA activity and is required for successful symbiosis. In this work, spontaneous symbiosis suppressors of Δ*cbrA* were identified as alleles of *divL* and *cckA*. In addition to rescuing symbiotic development, they restore wild-type cell cycle progression to free-living Δ*cbrA* cells. Biochemical characterization of the *S. meliloti* hybrid histidine kinase CckA *in vitro* demonstrates that it has both kinase and phosphatase activities. Specifically, CckA on its own has autophosphorylation activity, and phosphatase activity is induced by the second messenger c-di-GMP. Importantly, the CckA^A373S^ suppressor protein of Δ*cbrA* has a significant loss in kinase activity, and this is predicted to cause decreased CtrA activity *in vivo*. These findings deepen our understanding of the CbrA regulatory pathway and open new avenues for further molecular characterization of a network pivotal to the free-living cell cycle and symbiotic differentiation of *S. meliloti*.

**IMPORTANCE:**

*Sinorhizobium meliloti* is a soil bacterium able to form a nitrogen-fixing symbiosis with certain legumes, including the agriculturally important *Medicago sativa*. It provides ammonia to plants growing in nitrogen-poor soils and is therefore of agricultural and environmental significance as this symbiosis negates the need for industrial fertilizers. Understanding mechanisms governing symbiotic development is essential to either engineer a more effective symbiosis or extend its potential to non-leguminous crops. Here, we identify mutations within cell cycle regulators and find that they control cell cycle outcomes during both symbiosis and free-living growth. As regulators within the CtrA two-component signal transduction pathway, this study deepens our understanding of a regulatory network shaping host colonization, cell cycle differentiation, and symbiosis in an important model organism.

## INTRODUCTION

The establishment of a symbiotic relationship between nitrogen-fixing rhizobia and leguminous plants contributes to the global nitrogen cycle in an ecologically sustainable manner. Within host root nodules, rhizobia reduce N_2_ into ammonia to support plant growth in exchange for photosynthates, making this symbiosis of significant agricultural interest (1). *Sinorhizobium meliloti* is one well-studied model bacterium that participates in this symbiosis. It forms a chronic intracellular infection with various legumes, including *Medicago sativa* and *Medicago truncatula*, within a plant-derived organ called a nodule that forms on host roots (2, 3). During nodule infection, actively growing bacteria are guided into the nodule via an infection thread and eventually deposited into host cells. This process of infection likely occurs using the typical *S. meliloti* cell cycle observed during free-living reproduction (4). Intracellular bacteria then terminally differentiate into bacteroid cells capable of nitrogen fixation (4). A critical aspect of the bacteroid differentiation program involves significant changes to its cell cycle compared to free-living cells.

For *S. meliloti*, terminal differentiation into a bacteroid includes endoreduplication, resulting in an increased genome content via repeated rounds of DNA replication without cell division (4). This approximately 24N bacteroid then permanently exits the cell cycle, enlarged and branched in morphology, as a terminally differentiated G_0_ non-reproductive cell. This cell cycle program is controlled in part by host peptides synthesized within nodules and imported into bacterial cells (5, 6).

Nodule-specific cysteine-rich peptides also induce endoreduplication and block cell division *ex planta*, however, through unknown mechanisms, and so the precise mechanisms underlying *S. meliloti* cell cycle outcomes either *in planta* or *ex planta* remain unclear (7, 8).

*Caulobacter crescentus* is a model among alphaproteobacterial for understanding how DNA replication initiation is limited to once-and-only-once per cell cycle coupled to asymmetric cell division. In *C. cresecentus*, a two-component signal transduction (TCS) pathway regulates CtrA and thereby contributes to effectuating these cell cycle outcomes (9–11). CtrA is a DNA-binding response regulator whose activity is modulated by a complex TCS pathway through its phosphorylation and stability. It functions as a transcription factor that coordinates the temporal expression of cell cycle-related genes throughout the course of cell cycle progression. At cell division, it also contributes to asymmetric daughter-cell differentiation by binding near the chromosome origin to block DnaA-mediated initiation of DNA replication in swarmer daughter cells to impose a G_1_ phase cell fate. However, CtrA activity is absent from stalked daughter cells. This causes these cells to be born directly into S phase and able to immediately initiate a new round of DNA replication.

*S. meliloti* also couples DNA replication initiation with cell division to effectuate a once-and-only-once round of genome replication per cell division (4), along with asymmetric cell division to produce daughter cells with distinct replicative developmental fates (12). While CtrA helps to couple DNA replication initiation with cell division in *S. meliloti* (13, 14), it remains unclear how asymmetric daughter cell fate is established as there are no CtrA-binding sites near the chromosome origin to repress DNA replication initiation in G_1_ daughter cells as observed in *C. crescentus* (11).

**Fig 1:**
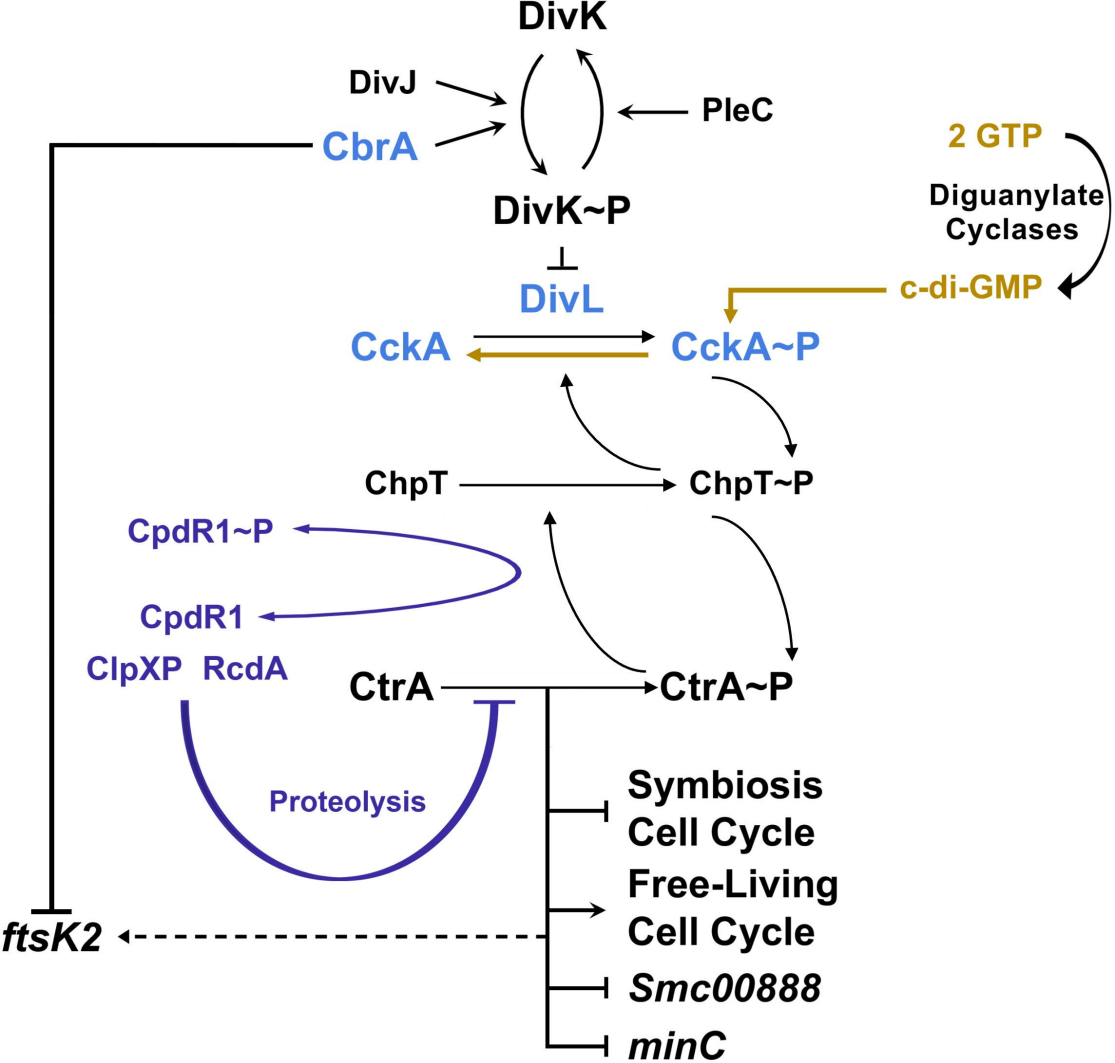
Model for CtrA-dependent cell cycle regulation in *S. meliloti*. DivJ and CbrA have been shown to function as DivK kinases, while PleC is a DivK phosphatase (11, 15, 16). When phosphorylated, DivK~*P* likely inhibits the ability of DivL to bind CckA and activate its kinase activity, thereby leading to the loss of CtrA~*P* activity. This TCS pathway regulating CtrA phosphorylation status is required to couple DNA replication initiation with cell division during free-living reproduction as well as bacteroid cell cycle differentiation during symbiosis (15, 16). CpdR1 is known to promote the proteolysis of CtrA via RcdA (highlighted in purple) (13, 14, 17). In addition, CckA phosphatase activity is enhanced in the presence of high concentrations of c-di-GMP, and this likely reduces the activity of CtrA downstream (highlighted in gold, this study). At present, the identification of *divL* and *cckA* symbiosis suppressors of Δ*cbrA* strongly supports the model that DivL and CckA act as a kinase/phosphate switch in conjunction with DivK to control the downstream phosphorylation status of CtrA (highlighted in blue, this study) through the histidine phosphotransferase ChpT. More specifically, the CckA^A373S^ suppressor protein is defective for kinase activity (this study), and it is, therefore, likely that repression of CtrA activity is critical to allowing symbiotic cell cycle differentiation. Downstream transcriptional changes to cell cycle and CtrA-regulated genes *Smc00888* and *minC* may contribute to the modulation of c-di-GMP levels and the process of cell division, respectively (this study). While CtrA indirectly affects the expression of *ftsk2* (14), and this cell cycle-regulated gene is transcriptionally repressed by CbrA in a manner independent of CtrA activity (this study).

*S. meliloti* has orthologs of many components of the *C. crescentus* CtrA TCS (Fig. 1) (11). Along with the DivK phosphatase PleC, which is essential in *S. meliloti* (15), there are two DivK histidine kinases (HKs): DivJ and the novel CbrA regulator (15, 16). Either Δ*divJ* or Δ*cbrA* is tolerated, however, the two genes are synthetically lethal (15), highlighting the critical role of regulating DivK phosphorylation for reproduction. The DivK/DivL/CckA phosphotransfer switch as well as the downstream target ChpT (11) are also present. Finally, while lacking PopA, *S. meliloti* does have CpdR1 and RcdA orthologs that regulate CtrA stability by mediating ClpXP proteolysis (13, 14, 17).

In *S. meliloti*, excess CtrA activity in either Δ*divJ* or Δ*cbrA* mutants results in cellular filamentation and polyploidy during free-living growth (15, 16). Importantly, these null mutants also have symbiotic defects that result in stunted plant growth. For Δ*cbrA*, suppression of this defect is observed with constitutive expression of non-phosphorylatable CpdR1^D53A^, which represses CtrA activity by promoting its proteolysis (Fig. 1) (13). Thus, it has been suggested that decreased CtrA activity may be important to allow bacteroid cell cycle differentiation.

While DivJ and CbrA components of the CtrA TCS are essential for symbiosis, the underlying mechanisms required to promote bacteroid cell cycle differentiation are not well understood. We utilized a forward-genetics approach by isolating spontaneous suppressors of the Δ*cbrA* mutant symbiosis defect, thus allowing the host to identify functions critical to bacteroid differentiation. Several suppressor alleles are located within either *divL* or *cckA*, whose activities are also able to suppress Δ*cbrA* free-living cell cycle defects and therefore represent universal functions within the *S. meliloti* TCS regulating cell cycle progression (Fig. 1). Importantly, the *cckA* suppressor mutation causes a significant loss of kinase activity *in vitro* and therefore represents the most direct evidence to date that downstream repression of CtrA activity is likely critical for effecting bacteroid cell cycle differentiation (Fig. 1).

## RESULTS

### Δ*cbrA* symbiosis suppressor mutations are in *divL* and *cckA*

**Fig 2:**
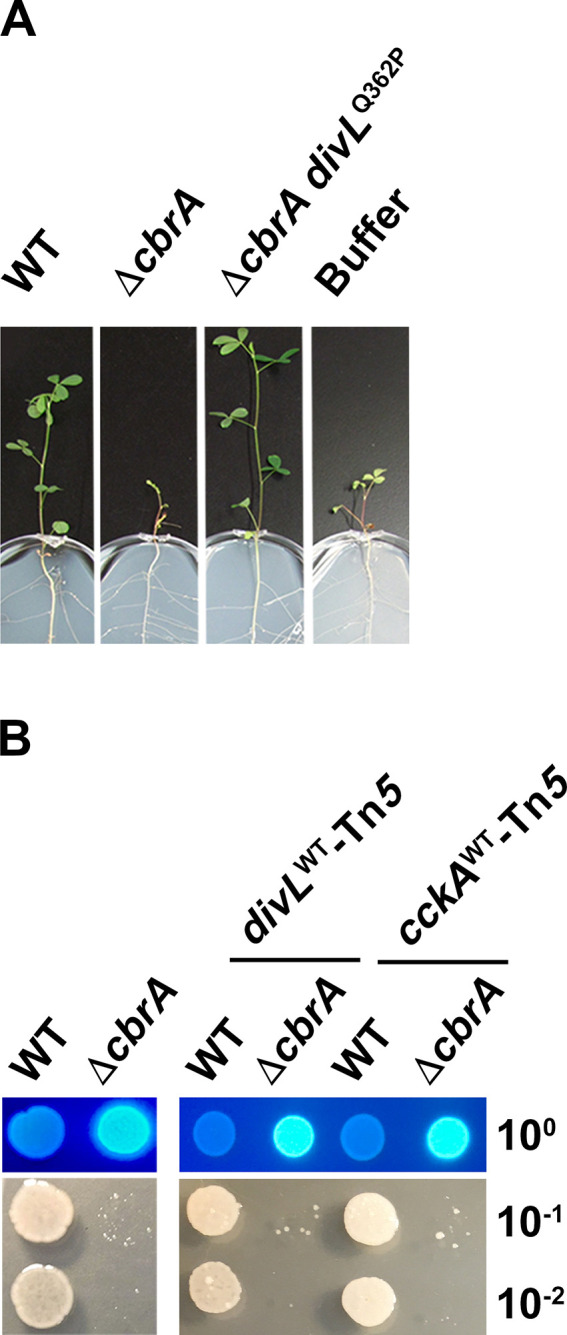
The Δ*cbrA* symbiosis defect can be suppressed by spontaneous second-site mutations. (**A**) Representative symbiosis results between *M. sativa* and one isolated symbiosis suppressor, later identified as Δ*cbrA divL*^Q362P^, in comparison to its Δ*cbrA* parent strain and wild type at 28 days post-inoculation. (**B**) The Δ*cbrA* mutant has pleiotropic free-living phenotypes that include succinoglycan overproduction and membrane instability. These phenotypes were assessed for the *divL*-linked (Tn*5-divL*) and *cckA*-linked (Tn*5-cckA*) transposons in an otherwise wild type or Δ*cbrA* background. Succinoglycan production was assayed using growth on calcofluor-supplemented media and visualized with UV light (top panel, 10° cell culture dilution), and membrane stability was assayed using growth on 10 mM DOC-supplemented media (bottom panel, 10^−1^ to 10^−2^ cell culture dilution). No detectable alteration of phenotype in either genetic background was observed for these and all other free-living phenotypes examined herein as well as symbiosis between *S. meliloti* and *M. sativa* (data not shown).

The Δ*cbrA* mutant has a severe symbiosis defect observed as underdeveloped white nodules unable to support *M. sativa* growth under nitrogen-limiting conditions due to the loss of nitrogen fixation (16). However, phenotypically wild-type nodules, elongated and pink, will rarely develop on these same plants. *S. meliloti* were isolated and cultured from each phenotypically wild-type nodule, and one colony per nodule was purified for further study. The absence of *cbrA* was confirmed using PCR for each isolate, and then its symbiotic phenotype was examined. Those found to provide a consistently improved outcome of pink nodule development and increased plant growth in comparison to the Δ*cbrA* parent strain were further studied (Fig. 2A).

**Fig 3:**
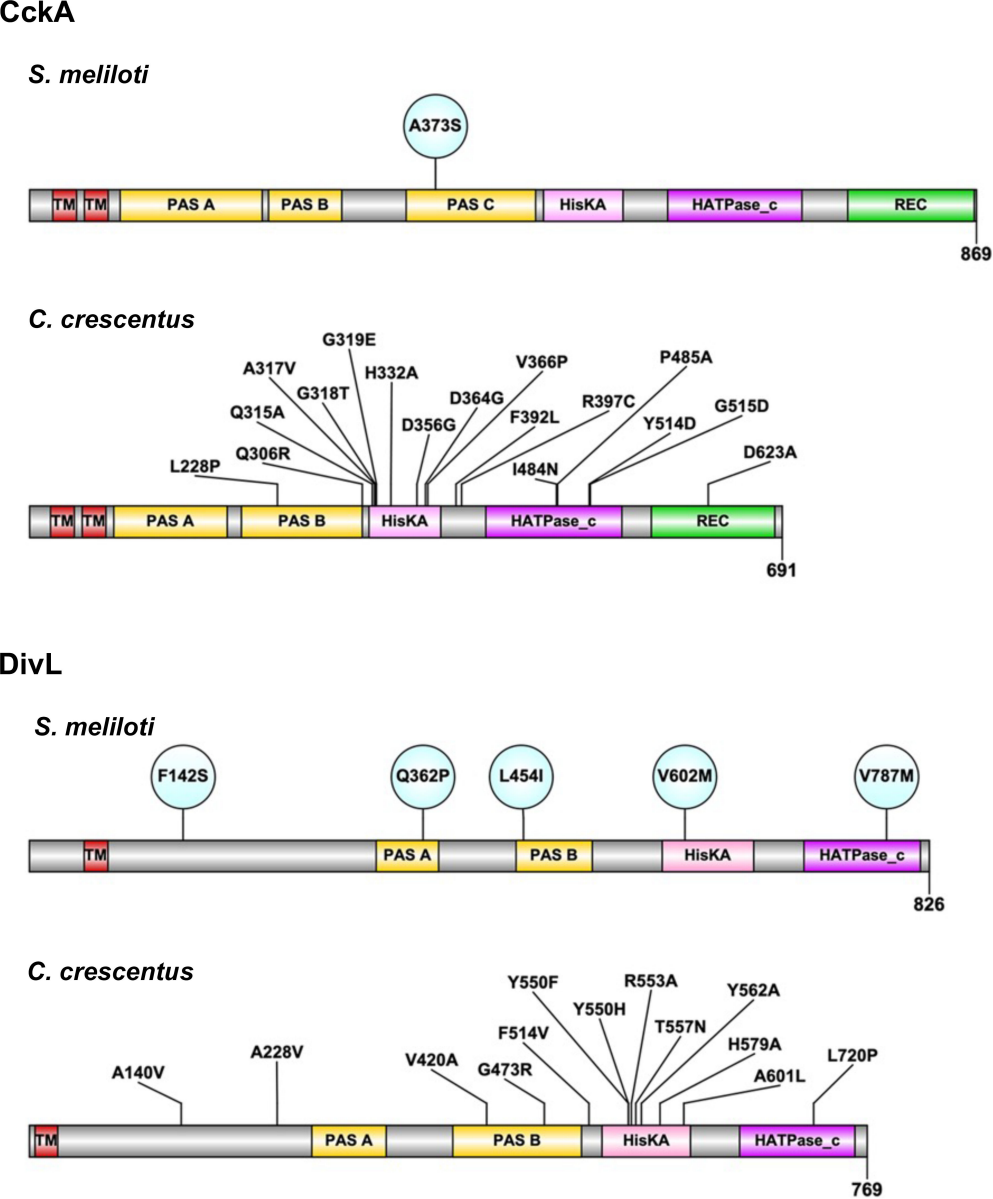
The predicted domain architecture of *S. meliloti* DivL and CckA with each Δ*cbrA* symbiosis suppressor allele indicated. Each *cckA* and *divL* mutation isolated as a symbiosis suppressor is indicated as a circle within the CckA and DivL domain structure (InterProScan). Each *C. crescentus* ortholog and relevant mutations that have been previously studied is included for comparison.

Whole-genome sequencing identified the precise genetic location of each Δ*cbrA* symbiosis suppressor mutation. For nine of these suppressors, a mutation is located within predicted components of the CtrA TCS: the atypical HK DivL and the hybrid histidine kinase CckA. Interestingly, three independently isolated suppressors contain the *cckA*^A373S^ allele, and as the only mutation within *cckA* identified, this suggests the affected activity is particularly important to symbiosis (Fig. 3). The domain architecture of *S. meliloti* CckA is similar to its ortholog in *C. crescentus* except for the prediction of an additional PAS (PER ARNT SIM) domain, between the transmembrane and HisKA domains. The PAS-C domain contains the *cckA*^A373S^ allele and aligns most closely with the PAS-B domain of *C. crescentus* CckA.

Six suppressors are located within *divL*, four of which are unique, while *divL*^V787M^ was independently isolated twice (Fig. 3). Mutations in *divL* are located throughout its ORF and each within a distinct domain, making it the most diverse target for suppressor mutations. *S. meliloti* DivL domain architecture is also similar to its *C. crescentus* ortholog with both having a transmembrane domain followed by two PAS domains, HisKA, and HATPase_C domains.

The allelic exchange was performed to confirm that each *divL* and *cckA* mutation is solely responsible for Δ*cbrA* suppression. To do this, a transposon linked to either *divL* or *cckA* was isolated. Free-living and symbiotic phenotypes were then assayed in both wild-type and Δ*cbrA* backgrounds, with and without each transposon. Since the presence of either transposon results in no observable phenotypic change compared to either parent strain (Fig. 2B), they were used to create *divL* and *cckA* single mutants as well as Δ*cbrA divL* and Δ*cbrA cckA* double mutants with clean genetic background. To simplify phenotypic descriptions below, rather than individually list each specific allele or mutant, we collectively refer to all *divL* and *cckA* mutants as “single mutants” and all Δ*cbrA divL* and Δ*cbrA cckA* mutants as “double mutants” when they share a phenotypic outcome, with any individual exceptions then identified.

**Fig 4:**
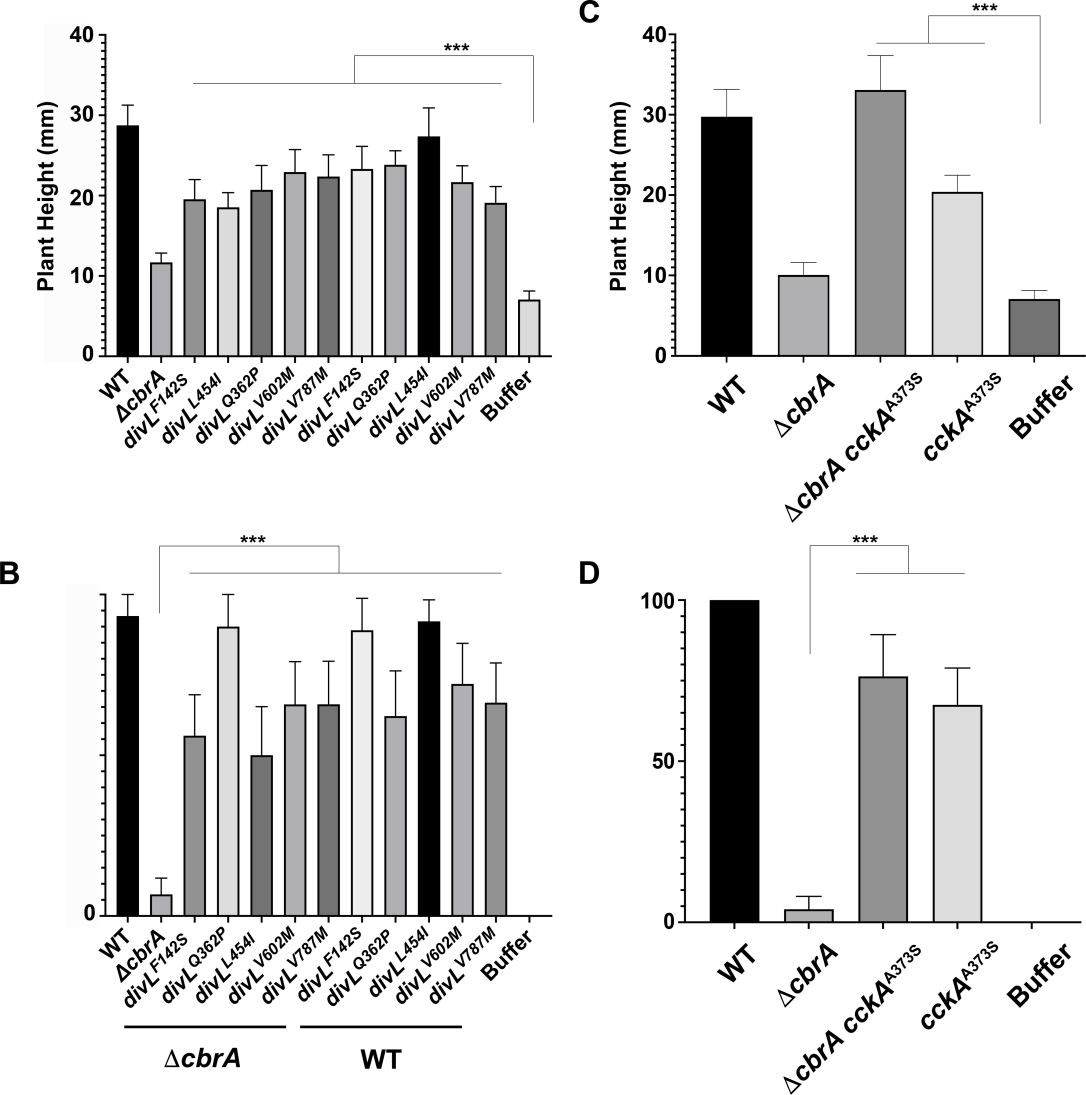
Suppressor alleles of *divL* and *cckA* improve the symbiotic outcome of *∆cbrA. M. sativa* plants were inoculated with *S. meliloti* and at 28 days post-inoculation plant height, and the percentage of phenotypically wild-type pink nodules was quantified (*N* = 10 plants per strain). (**A and C**) While the average height of plants inoculated with wild type is approximately 29 mm, the average height of plants inoculated with Δ*cbrA* is not significantly different from uninoculated (buffer control) plants (*P* = 0.9749) at approximately 10 mm. In contrast, the plant height of those inoculated with either a Δ*cbrA divL* or Δ*cbrA cckA* double mutant is significantly different from uninoculated (buffer control) plants (*P* < 0.0001) and more closely resembles those inoculated with wild type. (**B and D**) Regarding nodule development, plants inoculated with either a Δ*cbrA divL* or Δ*cbrA cckA* double mutant have a significantly increased percentage of at least 50% pink nodules in contrast to the Δ*cbrA* mutant rate of between 5%–10% (*P* < 0.0001). There is no significant difference in either (**A and C**) plant height or (**B and D**) pink nodule development in the *divL* and *cckA* single mutants when compared to the wild type. Statistical significance and *P* values for all comparisons were determined using a two-way ANOVA and Tukey Kramer test.

All *divL* and *cckA* suppressors rescue the Δ*cbrA* symbiosis for plant height and pink nodule formation as double mutants. Compared to uninoculated plants, Δ*cbrA* shows no significant difference in plant height (Fig. 4A and C). In contrast, Δ*cbrA* double mutants are significantly different from uninoculated plants and more closely resemble the wild type. Regarding nodule formation, Δ*cbrA* double mutants significantly increased the percentage of pink nodule development to ≥50% compared to Δ*cbrA* at 5%–10% (Fig. 4B and D). These observations are consistent with DivL and CckA functioning downstream of CbrA to regulate symbiosis. The symbiotic efficiency of each *divL* and *cckA* single mutant was also accessed and found not to be statistically different from the wild type (Fig. 4A and C).

### Succinoglycan overproduction and detergent sensitivity do not cause Δ*cbrA* symbiosis defects

**Fig 5:**
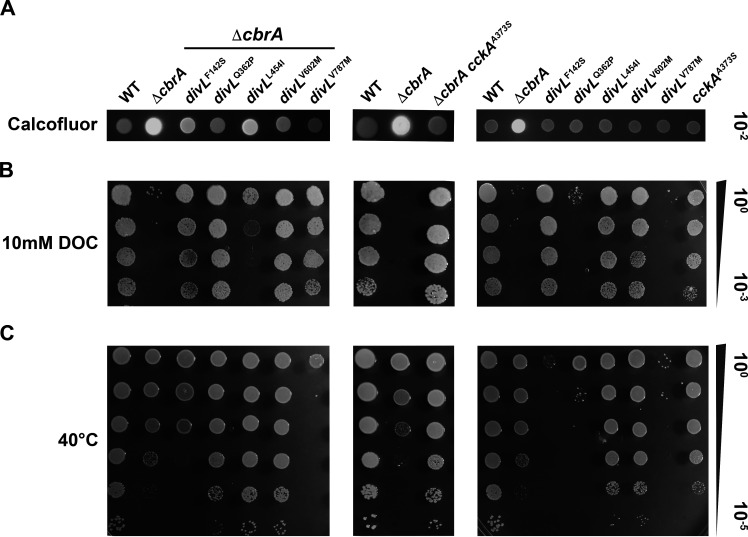
Free-living Δ*cbrA* succinoglycan overproduction and membrane instability phenotypes are suppressed by *divL* and *cckA* alleles. (A, left and middle panels) Succinoglycan production was assessed with calcofluor-supplemented media at 30°C using a serial-dilution spot assay and visualized by UV fluorescence. Wild type is calcofluor-dim, and Δ*cbrA* is calcofluor-bright. Δ*cbrA* double mutants display a decrease in calcofluor-fluorescence relative to the Δ*cbrA* parent strain to varying degrees with Δ*cbrA* divL^L454I^ having an intermediate phenotype. (A, right pane) Each *divL* and *cckA* single mutant displays a calcofluor-dim phenotype indistinguishable from wild type. (B, left and middle panels) Membrane stability was assessed with 10 mM DOC-supplemented media at 30°C using a serial-dilution spot assay. As a control, a replicate succinoglycan production assay with the same samples was performed (**A**), and no growth defects are observed in the absence of DOC. Wild type is DOC-resistant, and Δ*cbrA* is DOC-sensitive as seen by its inability to grow. Each Δ*cbrA* double mutant has increased DOC-resistance phenotype with the exception again of *divL*^L454I^, which displays an intermediate phenotype. (B, right panel) Each *divL* and *cckA* single mutant displays a DOC-resistance phenotype like wild type except for *divL*^Q362P^ and *divL*^V787M^, which exhibit a severe sensitivity to DOC nearly indistinguishable from Δ*cbrA*. (**C**) Growth was assessed at 40°C to determine whether any mutants exhibit a temperature-sensitive phenotype using a serial-dilution spot assay. As a control, a replicate succinoglycan production assay with the same samples was performed at 30°C (**A**), and no growth defects are observed at the standard temperature for ***S. **meliloti*. Δ*cbrA* grows nearly as well as wild type at 40°C, as do most Δ*cbrA* double mutants. Two exceptions are *divL*^V787M^ and to a lesser extent *divL*^F142S^. (C, right panel) Most *divL* and *cckA* single mutants grow nearly as well as wild type. Two exceptions are *divL*^V787M^ and *divL*^F142S^, which have a more severe temperature-sensitive phenotype in the presence of CbrA. In addition, *cckA*^A373S^ displays a mild temperature-sensitive growth defect in an otherwise wild-type background.

The exopolysaccharide succinoglycan is required for infection thread formation during early stages of host colonization, specifically the low molecular weight forms that are overproduced by Δ*cbrA* (18, 19). Thus, succinoglycan is not expected to be causative in Δ*cbrA* symbiosis defects, and therefore, suppressors specific to bacteroid differentiation may not impact this phenotype. To test this hypothesis, the relative production of succinoglycan was evaluated using media supplemented with calcofluor and visualized with UV-light. When succinoglycan production is assessed, wild type is calcofluor-dim, and Δ*cbrA* is calcofluor-bright (Fig. 5A). When compared to Δ*cbrA*, the *divL* and *cckA* double mutants display a decrease in calcofluor fluorescence relative to the parental strain, although to variable degrees, and therefore at least partially suppress Δ*cbrA* succinoglycan overproduction (Fig. 5A, left and middle panels).

However, the relatively weak suppression by *divL*^L454I^ indicates that this phenotype can be genetically separated from symbiosis outcome. Thus, succinoglycan overproduction is unlikely to be a primary factor in the Δ*cbrA* symbiotic defect given that Δ*cbrA divL*^L454I^ is symbiotically proficient (Fig. 4A and B). *divL* and *cckA* single mutants exhibit little to no observable effect on succinoglycan production (Fig. 5A, right panel).

The Δ*cbrA* mutant displays sensitivity to the detergent DOC, suggesting membrane instability for reasons that remain unclear (Fig. 5B) (18, 20). Each *divL* and *cckA* allele suppresses Δ*cbrA* DOC-sensitivity with wild-type growth with the notable exception of *divL*^L454I^, which displays an intermediate phenotype (Fig. 5B, left and middle panels). As with succinoglycan overproduction, this indicates that membrane instability is unlikely to be a primary factor in the Δ*cbrA* symbiotic defect given that Δ*cbrA divL*^L454I^ is symbiotically proficient (Fig. 4A and B). Most *cckA* and *divL* single mutants display wild-type growth on DOC with the exceptions of *divL*^Q362P^ and *divL*^V787M^, which exhibit DOC sensitivity similar to Δ*cbrA* (Fig. 5B, right panel). Unlike Δ*cbrA*, however, these mutants establish an effective symbiosis (Fig. 4A and B), further indicating that cell wall instability is unlikely to be a causative factor in the Δ*cbrA* symbiotic defect.

Growth at 40°C was used to assess mutants for temperature-sensitive (TS) growth as a potential outcome of cell cycle defects. Compared to wild type, Δ*cbrA* shows only a very mild growth defect (Fig. 5C). However, when combined with Δ*cbrA*, the *divL*^F142S^ and *divL*^V787M^ alleles cause noticeable TS growth defects (Fig. 5C, left panel), and these are even more severe in the wild-type background (Fig. 5C, right panel). Similarly, *divL*^Q362P^ displays a TS phenotype as a single mutant, but with Δ*cbrA*, this phenotype is negligible (Fig. 5C, left compared to right panel). Thus, the activity of these mutant proteins remains responsive to CbrA *in vivo* and therefore do not represent complete null alleles. Instead, they likely weaken a critical interaction, either intra- or intermolecular, that limits their ability to promote the minimal level of CtrA activity, which is essential in *S. meliloti*, required for reproduction.

### *divL* and *cckA* alleles identify core functions within the *S. meliloti* cell cycle pathway

**Fig 6:**
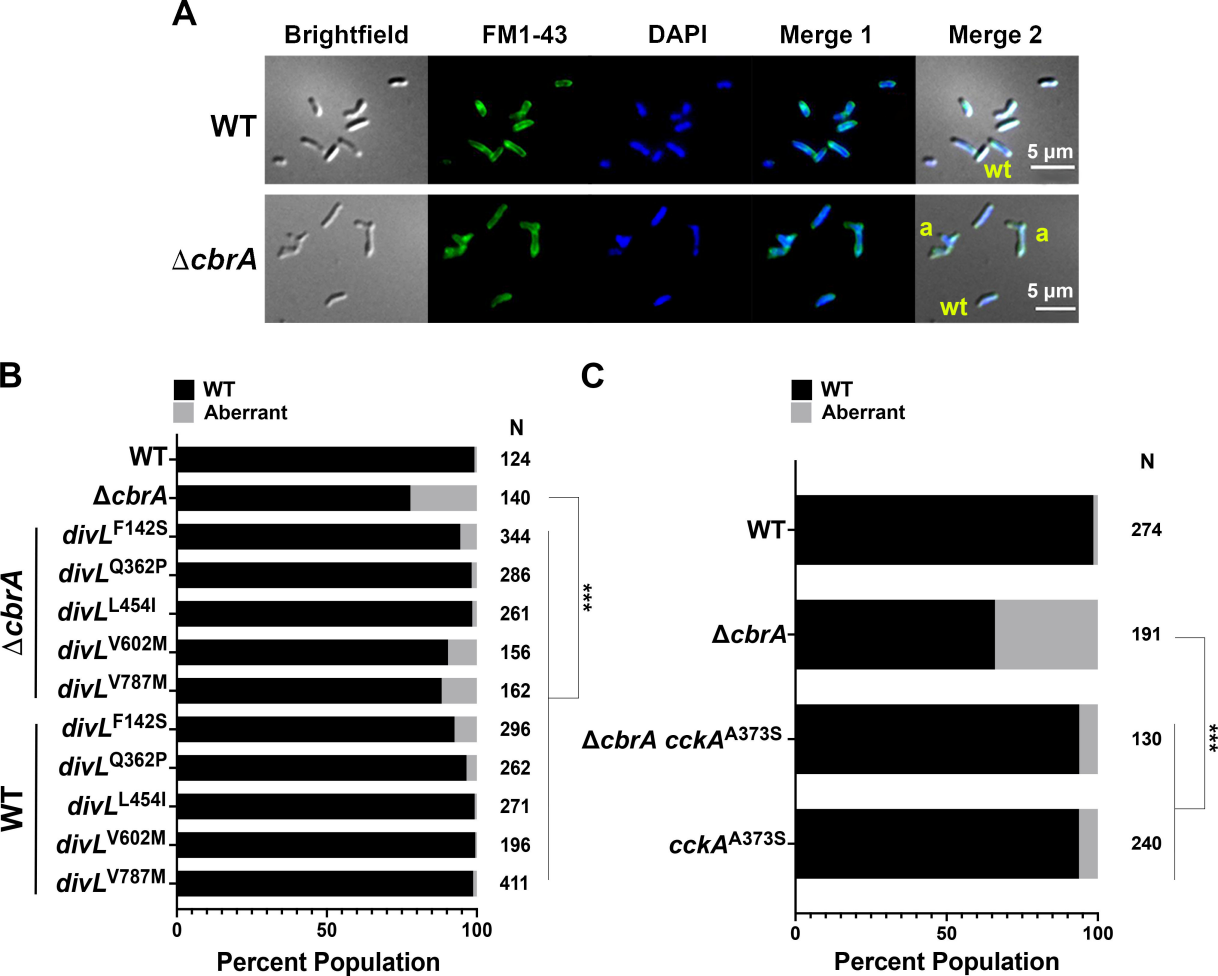
Free-living Δ*cbrA* cell morphology defects are suppressed by *divL* and *cckA* alleles. (**A**) Microscopy was performed on asynchronous logarithmic cultures to visualize cell morphology (brightfield DIC), cell membranes (FM1-43), and DNA (DAPI). The FM1-43 and DAPI images were combined (Merge 1), and all three images were combined (Merge 2) to highlight the filamentous cell morphologies observed. Example wild-type cells are labeled **wt** and filamentous cells are labeled **a**. (**B and C**) A population analysis was performed for cell morphology with *N* representing the number of cells examined for each strain. As a group, aberrant cells are those with either a length of >5 µm, those filamentous cells with three or more cell poles, and those that are minicell cocci with no observable cell pole. The wild type as well as all *divL* or *cckA* single and double mutants were found to be significantly different from Δ*cbrA* (*P* < 0.0001). Significance was determined using a two-way ANOVA and Tukey Kramer test.

As observed previously (16), Δ*cbrA* displays free-living cell cycle phenotypes of minicell production and filamentous morphology for approximately 30% of cells, whereas wild-type cells are bacilli with two cell poles measuring 1–2 µm in length (Fig. 6A).

Interestingly, minicells account for approximately 6% of the Δ*cbrA* cell population and are nearly 18% of all aberrant cells (data not shown), suggesting an underlying cell division defect. The *divL* and *cckA* suppressor alleles rescue Δ*cbrA* cell morphology defects to varying degrees with an aberrant cell population of ≤10% (Fig. 6B and C).

**Fig 7:**
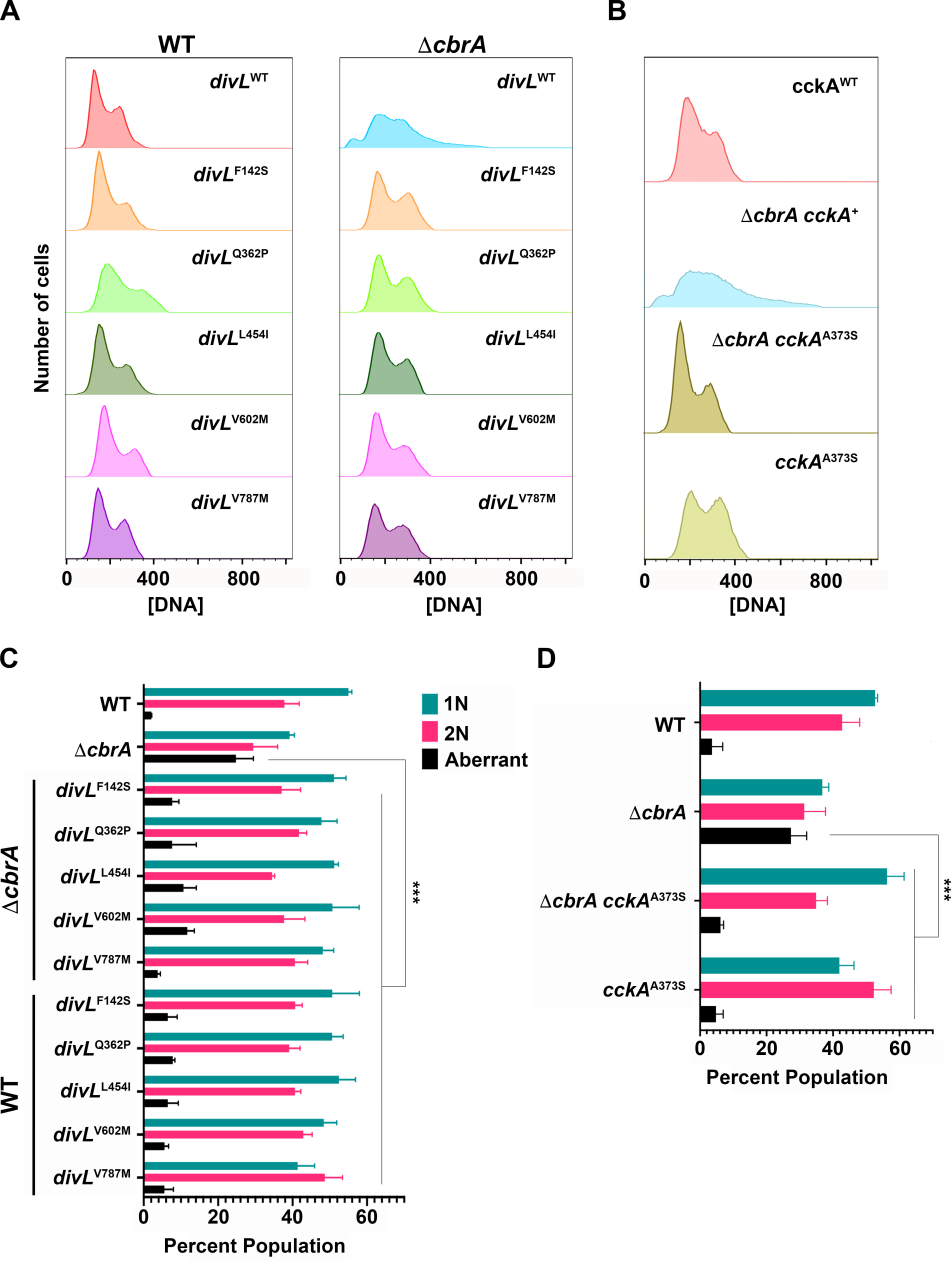
Free-living Δ*cbrA* polyploid defect requires wild-type DivL and CckA activity. Total cellular DNA was quantified by flow cytometry for cells growing asynchronously in the logarithmic phase with *N* = 3 independent biological replicates for all strains analyzed. One hundred thousand events were collected from each culture utilizing BD FACSAria II and subsequently analyzed using FlowJo. (**A and B**) Wild type displays a clear 1N and 2N peak of DNA, while Δ*cbrA* has in addition a <1N peak of minicells and >2N tail of polyploid cells. (**A**) Each *divL* and Δ*cbrA divL* double mutant is significantly different from Δ*cbrA* (*P* ≤ 0.0026) but not from the wild type. (**B**) The *cckA*^A373S^ and Δ*cbrA cckA*^A373S^ mutants are also significantly different from Δ*cbrA* (*P* = 0.0001) but not from the wild type. (**C and D**) The distribution of 1N, 2N, and aberrant (<1N and >2N) for cell populations growing asynchronously in the logarithmic phase was analyzed. In the Δ*cbrA* background, all *divL* and *cckA* alleles suppress the aberrant phenotypes observed in the Δ*cbrA* mutant (*P* < 0.0026). However, *divL*^V787M^ and *cckA*^A373S^ single mutants show a significant increase in the proportion of 2N cells (*P* = 0.0375 and 0.026, respectively). In addition, *cckA*^A373S^ shows a significant decrease in the number of 1N cells compared to wild type (*P* = 0.0162). Significance was determined using a two-way ANOVA and Tukey Kramer test.

*S. meliloti* initiates DNA replication once-and-only-once per cell cycle such that asynchronous logarithmic cell populations display two peaks of DNA content (4): G_1_ cells with 1N and G_2_ cells with 2N genome content (Fig. 7A and B). This strict coordination between DNA replication initiation and cell division is uncoupled in Δ*cbrA* with a significant population of polyploid cells having >2N genome content. The *divL* and *cckA* alleles restore wild-type cell cycle progression to Δ*cbrA* with 1N and 2N cell populations that lack both minicells (<1N) and polyploid cells (>1N; Fig. 6A and B). The mutations in *divL* and *cckA* therefore affect core cell cycle functions required for reproduction that are not unique to bacteroid cell cycle differentiation during symbiosis.

The distribution of cell populations falling into 1N, 2N, and aberrant genome content was quantified (Fig. 7C and D). Most *divL* single and Δ*cbrA* double mutants are not significantly different in DNA distribution compared to wild type (Fig. 7C). However, *divL*^V787M^ has an altered distribution with significantly more 2N cells than wild type (Fig. 7C). The *cckA*^A373S^ single mutant also has a significant reduction in the 1N cell population and a concurrent increase in the 2N cell population compared to wild type (Fig. 7D). These observations indicate that aberrant DivL^V787M^ and CckA^A373S^ mutant activity is associated with a mild G_2_ delay in cell division.

**Fig 8:**
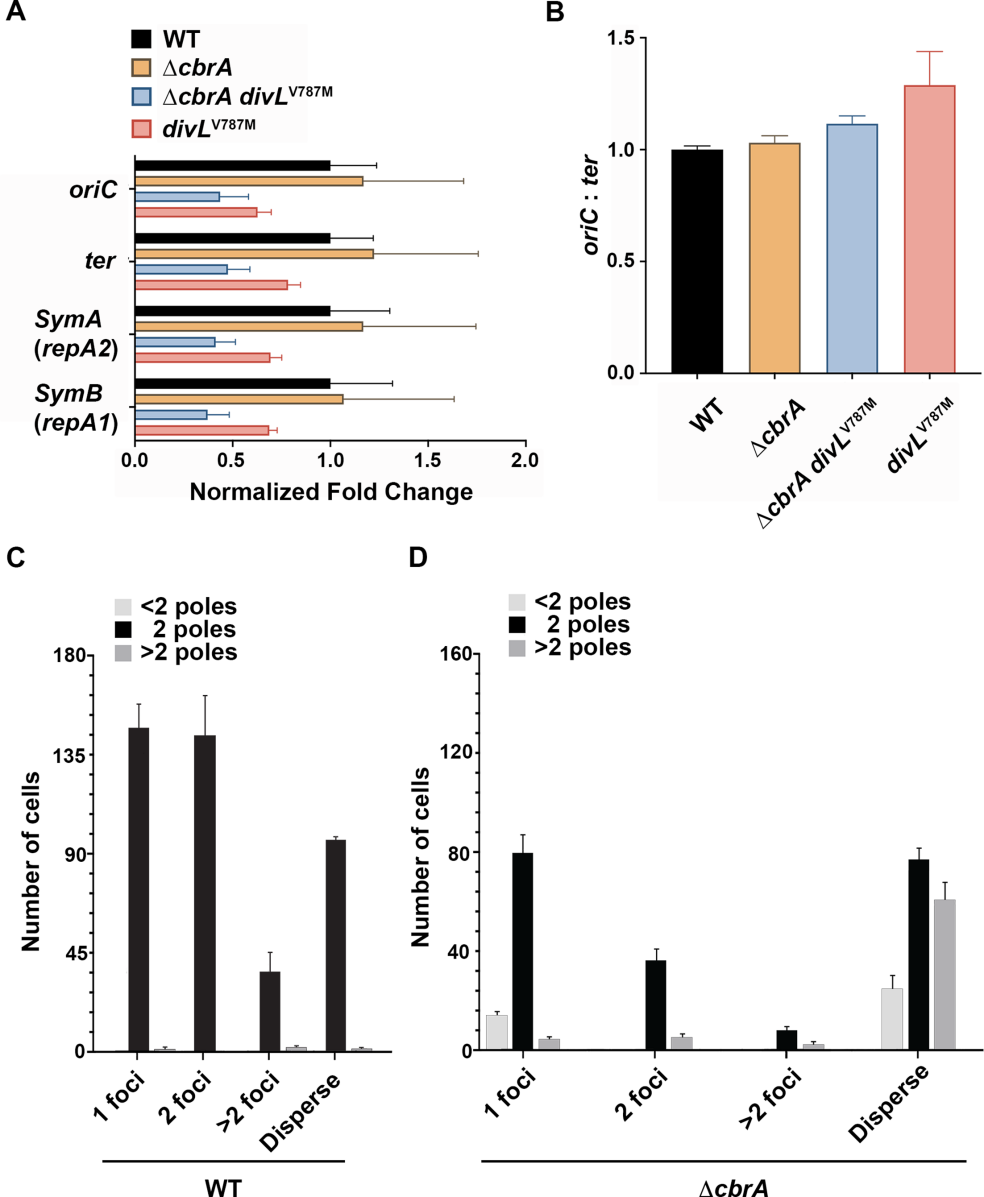
The Δ*cbrA* polyploid phenotype is not due to aberrant over-initiation of DNA replication initiation events. (**A**) RT-qPCR analysis was performed on whole-genome extractions from cells growing asynchronously in the logarithmic phase (*N* = 3 independent biological replicates for each strain). The 2^-ΔCT^ method was used for relative comparison between the four strains. The wild-type average value for each DNA target was normalized to 1. For each mutant strain, the average value for each DNA target was divided by the wild-type average value for that same target (*ori*, *ter*, *repA2*, and *repA1*). Similar results for pSymA and pSymB were obtained with repB and repC targets (data not shown). No comparisons among strains were found to be statistically significant using a two-way ANOVA. (**B**) The relative number of chromosome DNA replication initiation events was determined using the 2^−ΔCT^ method and represented as the *oriC:ter* ratio. No comparisons among the four strains were found to be statistically significant using one-way ANOVA. (**C and D**) DnaN-mCherry localization was examined in wild type (*N* = 427) and Δ*cbrA* (*N* = 311) cells growing in the logarithmic phase. Each cell was categorized based on the number of DnaN-mCherry foci, with active DNA replication reflected in cells with 2 or >2 foci and those preparing to start or end replication having a single focus. Cells with a dispersed phenotype do not have an active replisome. DnaN-mCherry localization in wild type and Δ*cbrA* populations was significantly different using the chi-squared test (*P* < 0.0001). Cells were further divided based on their morphology with those displaying a wild-type bacillus morphology having 2 poles, aberrant filamentous cells having >2 poles, and minicells represented as those with <2 poles.

To test whether Δ*cbrA* polyploidy results from additional DNA replication initiation events prior to cell division, qPCR was performed to measure relative chromosome origin (*oriC*) and terminus (*ter*) copy number. If the primary Δ*cbrA* defect is related to dysregulation of DNA replication with multiple initiation events at the *oriC* prior to completing replication at the *ter*, then it will have an increased *oriC* copy number relative to wild type. In contrast, if the primary defect is related to a delay in cell division, then its *oriC* copy number would remain wild type as a new replication initiation event would not occur until a prior round of chromosome replication is completed. For wild type, *oriC* and *ter* products were each normalized to 1, and mutants were then normalized to wild type. Interestingly, the *oriC* and *ter* copy number for Δ*cbrA* is indistinguishable from wild type (Fig. 8A), as are copy numbers for megaplasmids SymA and SymB (Fig. 8A). It was therefore not surprising that Δ*cbrA divL*^V787M^ and *divL*^V787M^ show no significant deviation from wild type for any replicon (Fig. 8A). Similarly, assessment of DNA replication initiation events through quantification of the *oriC:ter* ratio using the 2^−ΔCT^ method shows no statistically significant differences (Fig. 8B).

DnaN is a component of the replisome, so DnaN-mCherry was used as a proxy to visualize the number of replication events within individual cells growing in the logarithmic phase. *S. meliloti* cells in S phase will display a single focus that splits into two foci as one daughter chromosome is segregated to the opposite cell pole (12). Because of its tripartite genome, as cells transition through S phase, they display >2 foci as the symbiotic megaplasmids are replicated. For both wild type and Δ*cbrA*, cells were categorized based on their DnaN-mCherry phenotype: 1 focus, 2 foci, >2 foci, or a dispersed fluorescence phenotype (Fig. 8C and D). Active replication is represented by the presence of at least 1 focus, while cells with dispersed fluorescence are not actively replicating DNA as no replisomes are formed. The distribution of DnaN phenotypes is significantly different between wild type and Δ*cbrA* populations (*P* < 0.0001). Notably, Δ*cbrA* cells show an over-representation of cells that display a dispersed fluorescence pattern (23% ± 5% in wild type vs 51% ± 3% in Δ*cbrA*) and an under-representation of cells with foci (77% ± 5% in wild type vs 49% ± 3% in Δ*cbrA*). This observation further suggests that the Δ*cbrA* mutant experiences a significant delay in the timing of DNA replication initiation due either to an extended G_1_ or G_2_ phase, rather than excessive initiation during S phase.

Both wild type and Δ*cbrA* cells were additionally categorized based on the number of poles (Fig. 8C and D). As expected, the majority of wild type cells have 2 cell poles (99.7%) and display typical bacillus morphology regardless of DnaN phenotype. For Δ*cbrA* filamentous cells (>2 poles), the overwhelming majority (85%) exhibit a dispersed phenotype. These observations further suggest a significant delay in DNA replication initiation as cells with a dispersed phenotype lack an active replisome. This could result from either a G_1_ or a G_2_ arrest that delays re-entry into S phase and DNA replication initiation. Cumulatively, these observations demonstrate that the Δ*cbrA* polyploid phenotype is not caused by an aberrant increase in the number of DNA replication initiation events per cell cycle and instead likely reflects a role for CbrA in regulating cell division.

### CbrA regulates gene expression through two distinct pathways

**Fig 9:**
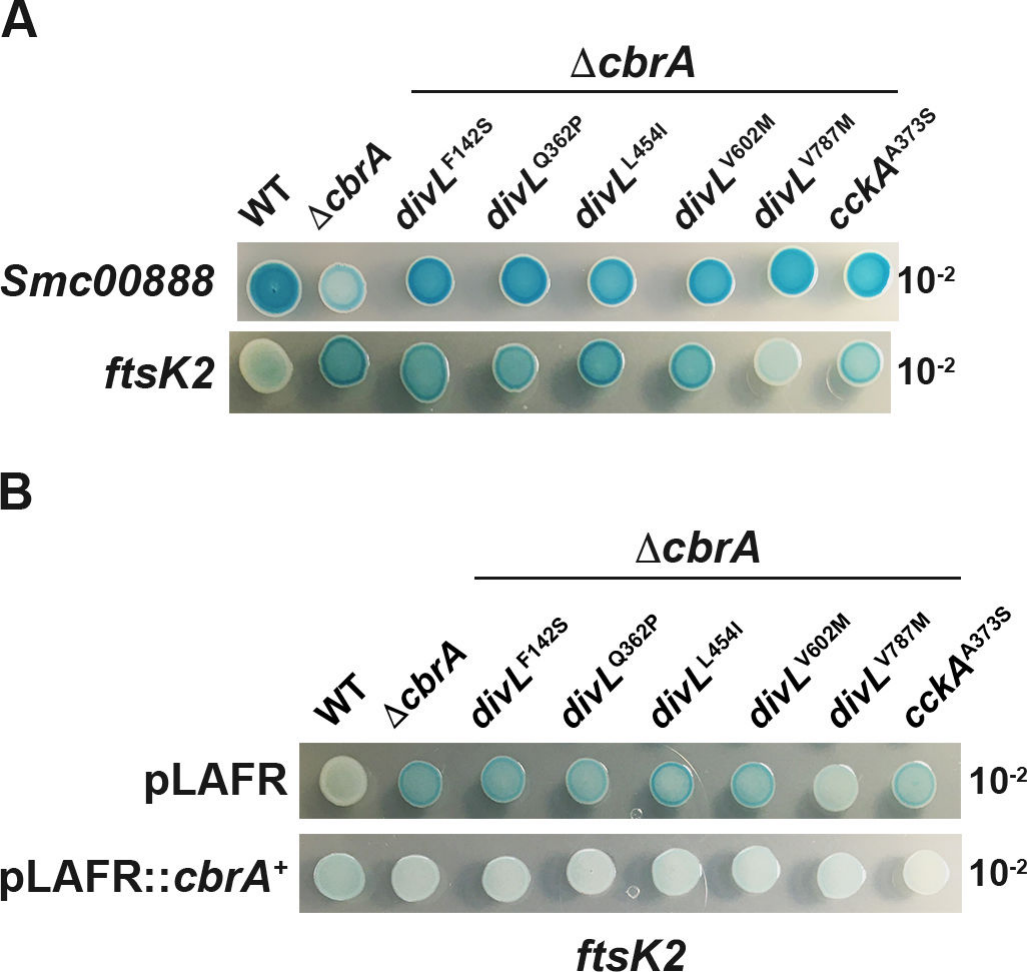
CbrA regulates cell cycle genes through two distinct pathways. Qualitative expression levels of an *Smc00888::GUS* and *ftsK2::GUS* transcription fusion were examined using a serial-dilution spot assay on LB/MC media supplemented with an X-GUS indicator dye. (A, top panel) Δ*cbrA* results in increased expression of *Smc00888::GUS* relative to wild type, and each *divL* and *cckA* allele restores wild-type expression levels. A similar pattern was observed for *Smc00887::GUS* expression; however, its overall level of activity is significantly decreased relative to *Smc00888::GUS* (data not shown). (A, bottom panel) The expression of *ftsK2::GUS* is increased in Δ*cbrA*; however, this phenotype is not restored to wild-type levels with the exception of *divL*^V787M^. (**B**) CbrA-dependence for *ftsK2::GUS* expression levels was examined through complementation analysis. (Top panel) With the pLAFR1 empty vector, high-level expression of *ftsK2::GUS* is observed in each mutant compared to wild type with the exception of Δ*cbrA divL*^V787M^. (Bottom panel) In contrast, the presence of pLAFR1::*cbrA*^WT^ restores wild-type expression in each mutant background.

CtrA plays a role in regulating cell cycle progression with both direct and indirect gene targets, some of which overlap with CbrA-regulated genes. Specifically, *Smc00888* and *Smc00887* are CbrA-regulated genes (20) subsequently identified as being CtrA-regulated (14). The expression of an *Smc00888::GUS* transcription fusion was therefore examined in wild type, Δ*cbrA*, and each double mutant. While *Smc00888::GUS* expression is decreased in Δ*cbrA*, each Δ*cbrA* double mutant rescues *Smc00888* expression to wild-type levels (Fig. 9A). This is consistent with each suppressor restoring cell cycle phenotypes to Δ*cbrA* by decreasing CtrA activity to wild-type levels.

Another gene identified as a direct target of CtrA-regulation is *minC* (14), and its overexpression leads to filamentous cell growth (21). Thus, it was of interest to examine *minC* expression as a gene expected to influence cell division. Unfortunately, the introduction of the *minC::GUS* transcription fusion into Δ*cbrA* presents with highly unstable colony phenotypes, perhaps because the fusion does disrupt normal *minCDE* expression (21), and was therefore not able to be studied further. Of note however, *minC::GUS* is stable in the wild type and in each of the Δ*cbrA* double mutants with qualitatively similar expression levels across these strain backgrounds (data not shown). Thus, the genetic instability of this *minC::GUS* fusion in Δ*cbrA* suggests that there is an additive disruption in cell division activities that impairs reproduction.

Δ*cbrA* is polyploid with filamentous growth resulting from a cell cycle defect independent from the licensing of DNA replication initiation (Fig. 7). FtsK2 is predicted to coordinate chromosome resolution with cytokinesis and is CtrA-regulated although in an indirect manner (14). An *ftsK2::GUS* transcription fusion was constructed to maintain *ftsK2* function and introduced into the wild type, Δ*cbrA*, and double mutant backgrounds. *ftsK2::GUS* expression increases in Δ*cbrA* compared to wild type; however, most *divL* and *cckA* alleles have little to no impact on this increased expression in Δ*cbrA* double mutants (Fig. 9A). An exception is Δ*cbrA divL*^V787M^, which decreases *ftsK2::GUS* expression relative to Δ*cbrA* (Fig. 9A). However, it appears that increased transcription of *fstK2* in Δ*cbrA* and its double mutants does not correlate with either aberrant morphology (Fig. 6B) or genome content (Fig. 7C).

To test whether increased *ftsK2* expression in Δ*cbrA* is due to the loss of *cbrA* or the loss of its adjacent genes, *fbpB* or SMc00777, complementation was performed.

The expression of *ftsK2::GUS* is unchanged with the pLAFR empty vector (Fig. 9A compared to B). In contrast, *ftsK2::GUS* expression is similar to wild type in each mutant background with pLAFR1::*cbrA*^WT^ (Fig. 9B). Thus, CbrA is responsible for increased *ftsK2::GUS* expression in Δ*cbrA* with most *divL* and *cckA* alleles unable to rescue this phenotype. While *ftsK2* was identified as an indirect transcriptional target of CtrA in a depletion experiment (14), these results suggest that the excess CtrA observed in Δ*cbrA* (13, 15, 16) is not the predominant transcription factor downstream of CbrA responsible for regulating *ftsK2* expression.

### Genetic characterization of *divL* and *cckA* suppressor alleles

In *C. crescentus*, DivL oscillates between direct interaction with either DivK~P, but not unphosphorylated DivK, or CckA (22, 23). This partner exchange regulates CckA localization to the new cell pole and its kinase/phosphatase switch within the CtrA TCS. Specifically, full CckA kinase activity requires DivL binding *in vivo*, and this interaction is blocked when DivK is phosphorylated and can titrate DivL away from CckA. Based on this *C. crescentus* model, CbrA as a DivK kinase will downregulate CckA kinase activity and thereby decrease CtrA activity (Fig. 1). In Δ*cbrA*, lower DivK~*P* levels would allow increased DivL binding to CckA, activating its kinase activity and thereby increasing CtrA activity (Fig. 1). Thus, DivL and CckA suppressor alleles of Δ*cbrA* are predicted to act through the downregulation of CckA kinase activity.

**Fig 10:**
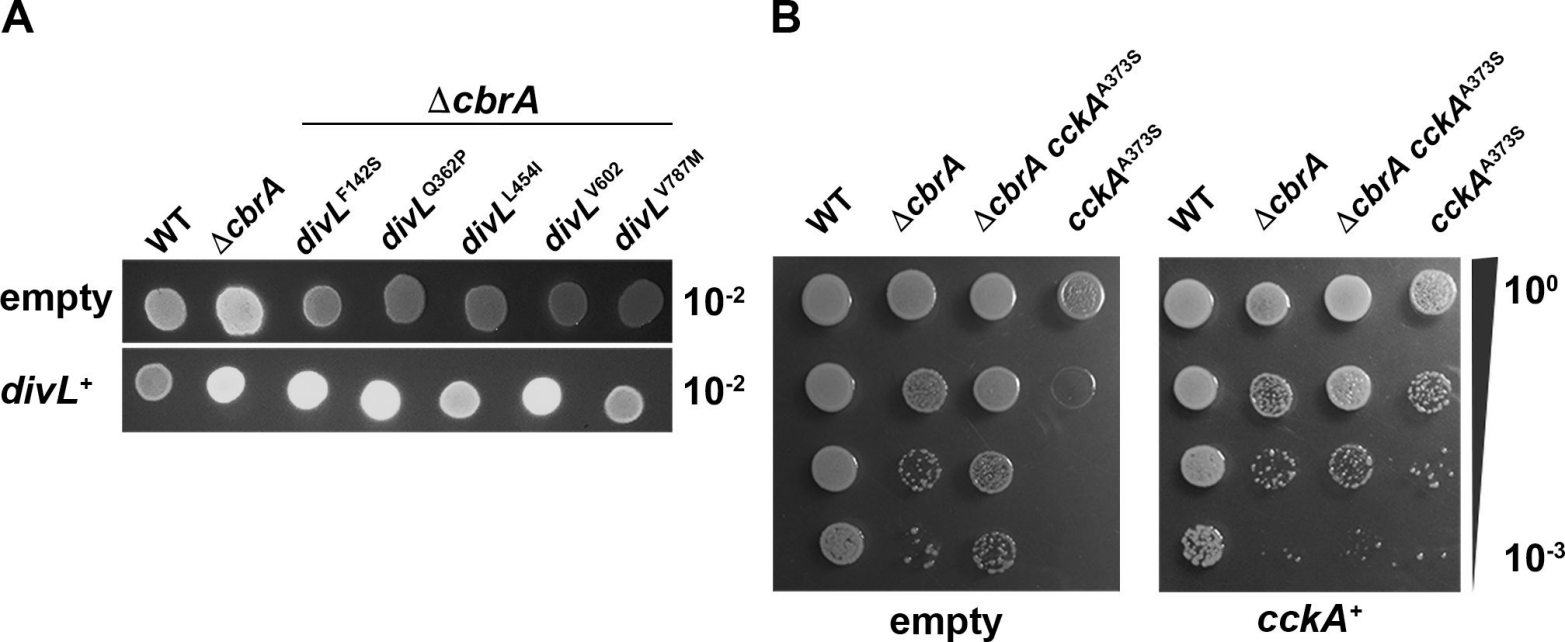
Complementation assay performed with *divL* and *cckA* alleles tests genetic relationship to the wild type. The low copy number pBBR1MCS-3 plasmid (empty) was used to create a complementation plasmid with either *divL*^WT^ or *cckA*^WT^ ORF and each with its native promoter region included as predicted by Softberry BPROM. Phenotypes for each strain were accessed using a serial-dilution spot assay on either (**A**) calcofluor-supplemented LB media grown at 30°C or (**B**) on LB media grown at 40°C. (A, top row) In the presence of pBBR1MCS-3, the wild type has a calcofluor-dim phenotype, while Δ*cbrA* is calcofluor-bright. Each Δ*cbrA divL* double mutant has a calcofluor-dim phenotype relative to Δ*cbrA* in the presence of pBBR1MCS-3. (A, bottom row) In the presence of pBBR1MCS-3::*divL*^WT^, the wild type maintains a calcofluor-dim phenotype, while Δ*cbrA* remains calcofluor-bright. The *divL*^F142S^, *divL*^Q362P^, and *divL*^V602M^ alleles are fully complemented by pBBR1MCS-3::*divL*^WT^ to the Δ*cbrA* calcofluor-bright phenotype, which indicates these alleles are recessive. The intermediate calcofluor-bright phenotype of Δ*cbrA divL*^L454I^ in the presence of pBBR1MCS-3 *divL*^WT^ indicates this allele is semi-dominate, while the calcofluor-dim phenotype of *divL*^V787M^ shows that it is dominant. (B, left) During growth at 40°C with pBBR1MCS-3, *cckA*^A373S^ displays a significant TS growth defect in contrast to Δ*cbrA* and Δ*cbrA cckA*^A373S^ mutants. (B, right) During growth at 40°C with pBBR1MCS-3::*cckA*^WT^, *cckA*^A373S^ growth is now similar to other mutant backgrounds, Δ*cbrA* and Δ*cbrA cckA*^A373S^, which indicates this allele has been complemented and is recessive.

To genetically characterize each *divL* allele, pBB1RMCS-3::*divL*^WT^ was introduced into Δ*cbrA divL* double mutants, and succinoglycan production was assayed as an output for complementation. Each Δ*cbrA divL* mutant displays a wild-type calcofluor-dim phenotype relative to Δ*cbrA* with the pBB1RMCS-3 empty vector (Fig. 10A, top row). Several Δ*cbrA divL* mutants with pBBR1MCS-3::*divL*^WT^ display a calcofluor-bright phenotype similar to Δ*cbrA* (Fig. 10A, bottom row). Thus, these alleles are recessive to *divL*^WT^ and may either reduce its CckA binding or increase its affinity for DivK/DivK~*P* (Fig. S1), resulting in an overall decrease in CtrA activity in the Δ*cbrA* background. In contrast, Δ*cbrA divL*^L454I^ displays an intermediate calcofluor phenotype with pBBR1MCS-3::*divL*^WT^ and has only partial complementation, while Δ*cbrA divL*^V787M^ displays a calcofluor-dim phenotype like wild type and is therefore dominant (Fig. 10A, bottom row). Thus, DivL^L454I^ and DivL^V787M^ can likely bind CckA and thereby prevent full complementation by DivL^WT^. For both alleles, it appears they are either unable to effectively promote CckA kinase activity or aberrantly enhance its phosphatase activity upon binding (Fig. S1), thereby decreasing CtrA activity in Δ*cbrA*.

**Fig 11:**
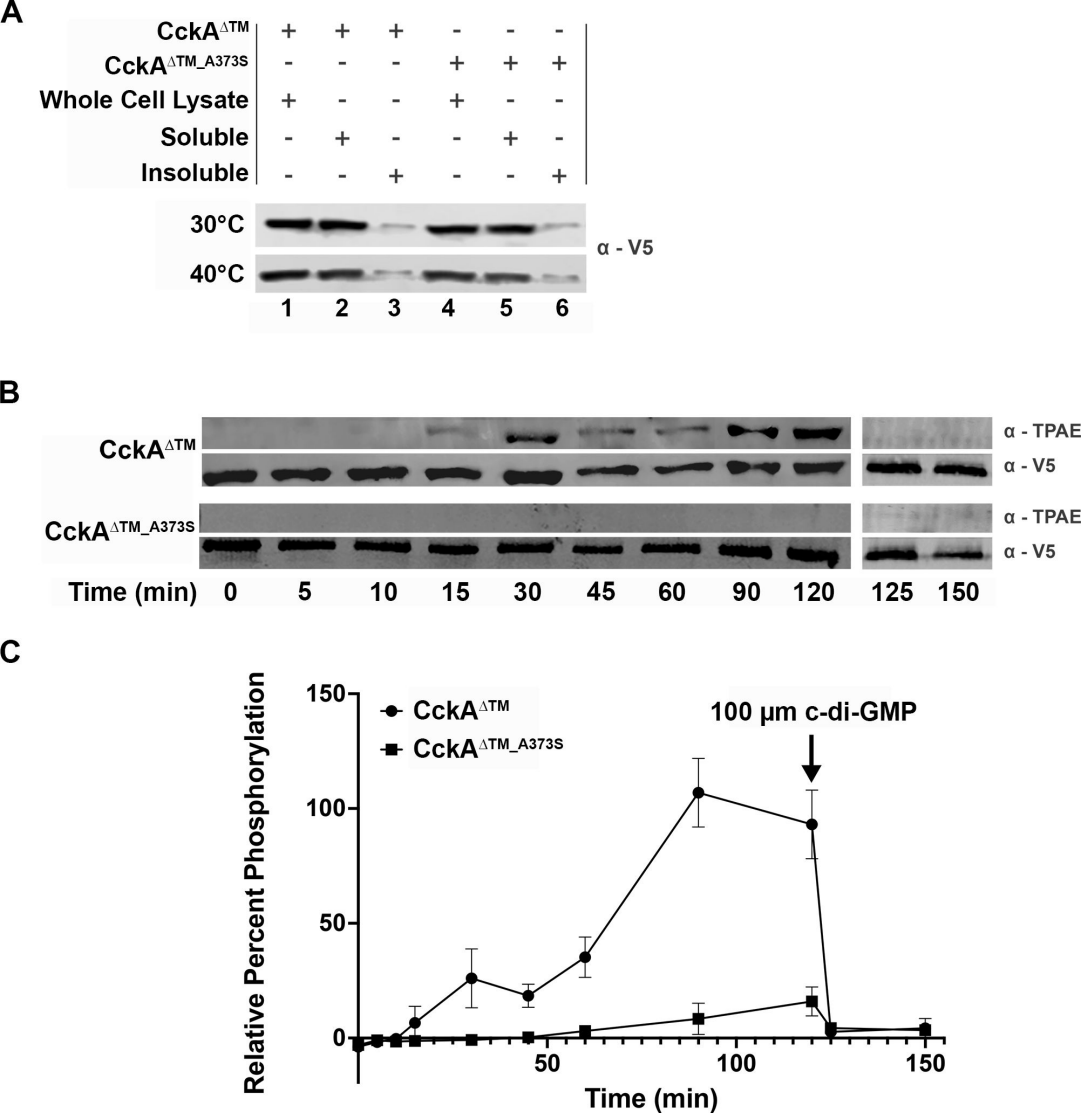
*S. meliloti* CckA^ΔTM^ displays kinase activity and responds to c-di-GMP with a switch to phosphatase activity. (**A**) V5 affinity-tagged CckA^ΔTM^ and CckA^ΔTM_A373S^ proteins were expressed in BL21 grown at 30°C and 40°C. Both protein variants are expressed (lanes 1 and 4) and soluble (lanes 2 and 5) with minimal insoluble protein (lanes 3 and 6) at both growth temperatures. (**B**) V5-CckA^ΔTM^ (row 1, α-TPAE phosphorylated protein; and row 2, α-V5 total protein) and V5-CckA^ΔTM_A373S^ (row 3, α-TPAE phosphorylated protein; and 4, α-V5 total protein) were incubated with ATPγS in a kinase assay for 120 min with samples taken at the indicated time points. V5-CckA^ΔTM^ autophosphorylation is detectable at 15 min (lane 4), while V5- CckA^ΔTM_A373S^ autophosphorylation is imperceptible until 60 min when a weak signal becomes detectable (lane 7). At 120 min, 100 µm c-di-GMP was added to each kinase reaction to test for phosphatase activity. For V5-CckA^ΔTM^, complete dephosphorylation is observed within 5 min at the 125 min timepoint. (**C**) For each sample, the average amount detected with α-TPAE was normalized to the average total protein detected with α-V5 at each time point to quantify the percentage of phosphorylated protein. The V5-CckA^ΔTM^ value was maximal at 90 min and therefore normalized to 100%. V5-CckA^ΔTM_A373S^ autophosphorylation activity is significantly diminished compared to V5-CckA^ΔTM^ from 30 min onward (*P* < 0.01). *N* = 3 independent biological replicates for all enzymatic assays. Significance was determined using a two-way ANOVA and Tukey Kramer test.

In an otherwise wild-type background, pBBR1MCS-3::*cckA*^WT^ results in an aberrant calcofluor-bright phenotype (data not shown), and therefore, complementation could not be tested using this output. Instead, *cckA*^A373S^ complementation was performed using TS growth of the *cckA*^A373S^ single mutant as the pBBR1MCS-3 empty vector exacerbated this otherwise weak phenotype (Fig. 5C, middle and right panels; and Fig. 10B, left panel). Importantly, CckA^A373S^ remains responsive to CbrA signaling at 40°C (Fig. 5C, middle and right panels; Fig. 10B, left panel), and both CckA^WT^ and CckA^A373S^ are equally expressed and soluble at both 30°C and 40°C (Fig. 11A). Thus, the TS phenotype used to evaluate complementation is not due to instability or unfolding of CckA^A373S^ that would result in null activity.

Growth of each mutant strain is less robust than wild type either with the pBBR1MCS-3 empty vector or the pBBR1MCS-3::*cckA*^WT^ complementation vector (Fig. 10B), indicating that each has a less efficient cell cycle supporting reproduction.

However, *cckA*^A373S^ with pBBR1MCS-3 displays very limited growth at 40°C (Fig. 10B, left panel). Importantly, *cckA*^A373S^ pBBR1MCS-3::*cckA*^WT^ grows as well as the other mutant strains, Δ*cbrA* and Δ*cbrA cckA*^A373S^, when combined with pBBR1MCS-3::*cckA*^WT^ (Fig. 10B, right panel). This complementation indicates that the *cckA*^A373S^ allele is recessive and attenuating Δ*cbrA* phenotypes through either decreased binding to DivL or an inherently decreased kinase activity even when bound to DivL (Fig. S1).

### CckA^A373S^ is significantly impaired for kinase activity

The soluble cytoplasmic portion of CckA and CckA^A373S^ proteins was V5-tagged for purification and enzymatic characterization. As the single mutant *cckA*^A373S^ exhibits a weak TS phenotype when grown at 40°C, V5-CckA^ΔTM^ and V5-CckA^ΔTM_A373S^ were expressed at both 30°C and 40°C. Western blot analysis shows that both proteins are equally stable and located primarily within the soluble cell fraction at both temperatures (Fig. 11A). This, combined with no additive TS phenotype as a Δ*cbrA cckA*^A373S^ double mutant compared to Δ*cbrA* (Fig. 5C, middle and right panels; Fig. 10B, left panel), strongly suggests that CckA^A373S^ retains some functional activity and is not an unstable or unfolded protein *in vivo*. Thus, the V5-CckA^ΔTM_A373S^ protein can be assessed for *in vitro* enzymatic activities compared to V5-CckA^ΔTM^.

CckA^A373S^ is complemented by CckA^WT^ (Fig. 10B), suggesting it has lost the ability to either bind DivL or perform kinase activity (Fig. S1). If CckA^A373S^ kinase activity is decreased relative to CckA^WT^, then this would be reflected in an *in vitro* enzyme assay. The non-radioactive ATP analog ATP-γ-S was used in kinase assays, as one advantage is its increased stability to spontaneous hydrolyzation, and phosphorylated protein was quantified by western blot using antibodies to the thiophosphate ester produced by post-reaction alkylation. Proteins were purified using their V5 tag and then incubated with ATP-γ-S over the course of 2 hours. Samples at the indicated timepoints were probed by western blot for phosphorylated protein (α-TPAE) and total protein (α-V5; Fig. 11B and C). CckA^ΔTM^ exhibits autophosphorylation within 15 min and displays increasing phosphorylation over time. In contrast, CckA^ΔTM_A373S^ does not demonstrate detectable autophosphorylation activity, and at 2 hours, the total concentration of phosphorylated protein is significantly decreased relative to CckA^ΔTM^. Together, these results show CckA^ΔTM_A373S^ is defective for autophosphorylation *in vitro* and is consistent with the complementation of *cckA*^A373S^ *in vivo* reflecting its activity as a recessive allele (Fig. 10B).

In *C. crescentus*, CckA has been shown to bind c-di-GMP, and this enhances its switch to phosphatase activity (23–26). To test whether this regulation may also be present in *S. meliloti*, c-di-GMP was added to each kinase reaction at 120 min, and samples were subsequently taken at 125 and 150 min (Fig. 11B and C). Within 5 min of ci- di-GMP addition, minimal phosphorylated CckA^ΔTM^ is detectable, showing c-di-GMP can bind this protein and enhance its phosphatase activity. Since the level of phosphorylated CckA^ΔTM_A373S^ at 120 min is nearly undetectable, it is unclear whether the addition of c-di-GMP causes a further decrease. Overall, these data show c-di-GMP may be a regulator of the *S. meliloti* CtrA TCS pathway *in vivo* by binding CckA and enhancing its phosphatase activity.

## DISCUSSION

CbrA functions as a DivK kinase to regulate CtrA activity during free-living growth (13, 15, 16), and this study represents a forward-genetic screen for mutations that suppress the severe symbiotic deficiency of Δ*cbrA* in order to gain insight into the molecular requirements underlying bacteroid cell cycle differentiation. CtrA levels are decreased in bacteroids compared to free-living cells, which indicates that this may be a requirement for symbiosis. Consistent with this, Δ*cbrA* and Δ*divJ* mutants have increased CtrA during free-living growth and are unable to establish an effective symbiosis (15, 16). The identification of symbiosis suppressors in *divL* and *cckA* further supports the hypothesis that the Δ*cbrA* defect is due, at least in part, to the mis-regulation of CtrA. Significant TS growth defects are observed for several *divL* alleles, and in particular, the strong TS phenotype of *divL*^V787M^ as both a single and double mutant indicates that *divL* is an essential gene in *S. meliloti* (Fig. 5C), as in *C. crescentus*, and possibly due to the requirement for minimal CtrA activity to support reproduction during free-living growth.

Δ*cbrA* has pleiotropic free-living phenotypes, including succinoglycan overproduction and membrane sensitivities, in addition to cell cycle phenotypes of filamentous growth and polyploidy (16, 18). While retaining moderate succinoglycan overexpression and DOC-sensitivity (Fig. 5A and B), *divL*^L454I^ restores symbiosis to Δ*cbrA* (Fig. 4A and B). Importantly, it also restores free-living cell cycle progression to Δ*cbrA* (Fig. 6B, 7A and C). Moreover, *divL*^Q362P^ and *divL*^V787M^ single mutants display a strong DOC-sensitive phenotype (Fig. 5B) while maintaining free-living cell cycle progression and the ability to establish an effective symbiosis (Fig. 4A, B, 6C, 7B and D). These observations underscore the relative importance of the role CbrA plays in regulating cell cycle outcomes as the critical requirement for symbiosis .

For *S. meliloti*, DNA replication initiation is strictly limited to once-and-only-once per cell division (4); however, Δ*cbrA* has a >2N polyploid phenotype (Fig. 7). Our data demonstrate this phenotype is not caused by an increased rate of DNA replication initiation (Fig. 8) and instead points toward a cell division defect. Although many CtrA-regulated genes remain uncharacterized, this conclusion is consistent with the identification of *minCDE*, along with cell envelope functions, as transcription targets (14). While misexpression of either DnaA or its regulator, Hda, can alter the rate of chromosomal DNA replication initiation in *S. meliloti* (12), neither of these genes has been identified as CtrA-regulated (14). Consistent with this, while CtrA depletion and the hyperactivation of CtrA activity in Δ*cbrA* result in filamentous polyploid cells (Fig. 7) (16), these cells maintain a wild-type copy number for all replicons (Fig. 8). This contrasts with the *C. crescentus divK* null phenotype, which exhibits a strong G1 arrest with 1N cells due to the hyperactivation of CtrA, thereby blocking DnaA replication initiation at the origin (27, 28). Thus, it remains unclear whether, and if so how, CtrA regulates DNA replication initiation or the establishment of daughter cell replicative asymmetry in *S. meliloti*.

The only *cckA* symbiosis suppressor allele recovered was *cckA*^A373S^ within its PAS-C domain (Fig. 3), although it was independently isolated three times and therefore reflects the importance of this residue for regulating CckA activity during bacteroid differentiation. Moreover, this allele is the most effective symbiosis suppressor as reflected in plant growth and pink nodule formation (Fig. 4C and D). It also restores wild-type phenotypes to the Δ*cbrA* mutant during free-living growth, including cell cycle progression (Fig. 6C, 7B and D) and CtrA-regulated gene expression (Fig. 9A).

Complementation analysis further reveals that *cckA*^A373S^ is a recessive mutation (Fig. 10B), and consistent with this, the purified CckA^ΔTM_A373S^ protein displays significantly reduced autophosphorylation activity *in vitro* (Fig. 11B and C). These observations combined reinforce prior observations (13) and further indicate that decreasing the flow of phosphate toward CtrA likely plays a critical role in allowing bacteroid cell cycle differentiation.

For CckA^A373S^, the most closely aligned domain in *C. crescentus* is PAS-B (Fig. 3). This *C. crescentus* PAS-B domain is predicted to interact closely with its own DHp- CA catalytic core based on structural analyses and is also needed for responses to DivL and c-di-GMP (24, 26, 27, 29–31). Given the role that c-di-GMP plays in modulating CckA activity in *C. crescentus*, it was of interest to determine whether this signaling molecule could regulate *S. meliloti* CckA as well. When c-di-GMP is added to maximally phosphorylated CckA^ΔTM^, a rapid rate of dephosphorylation is observed (Fig. 11B and C). Thus, *S. meliloti* CckA can bind this important signaling molecule, and binding enhances its phosphatase activity *in vitro*.

In *C. crescentus*, DivL is required to ensure the proper localization of CckA and enhance its kinase activity, which subsequently leads to downstream phosphorylation of CtrA (22). The PAS domains of DivL have been identified as regions required for CckA binding, while the HisKA and CA domains of DivL have been proposed to be docking surfaces for DivK~*P* (22, 23, 25, 29, 31). Two PAS domain mutations were isolated as suppressors of a *C. crescentus*Δ*divJ* mutant: *divL*^V420A^ and *divL*^G473R^ (32) (Fig. 3). They suppress the cell cycle defects of a Δ*divJ* mutant while simultaneously conferring TS sensitivity, particularly in the wild-type background, by decreasing CtrA~*P* levels.

Notably, *C. cresentus* DivL^A601L^ (Fig. 3) stimulates CckA kinase activity while abolishing its interaction with DivK~*P* (22, 29). It is therefore not surprising that four of the five isolated *S. meliloti divL* suppressors are located within these domains (Fig. 3), presumably modulating direct interaction with either DivK/DivK~*P* or CckA and thereby affecting CtrA activity.

Complete complementation of *divL*^F142S^, *divL*^Q362P^, and *divL*^V602M^ suggests they suppress Δ*cbrA* phenotypes through a loss of interaction with CckA or an increased affinity for either DivK or DivK~*P* (Fig. 10A; Fig. S1). Conversely, *divL*^L454I^ and *divL*^V787M^ exhibit incomplete or no complementation (Fig. 10A), respectively, suggesting they are capable of binding CckA but have lost the ability to effectively promote CckA kinase activity or enhance phosphatase activity as a binding partner (Fig. S1). It will be of significant interest to further characterize each of these mutant proteins to test these hypotheses and gain insight into the molecular functions of DivL in *S. meliloti* that are required for cell cycle progression.

## MATERIALS AND METHODS

### Cell culture techniques

*Escherichia coli* strains were grown at 37°C in LB, and *S. meliloti* strains were grown at 30°C in LB containing 2.5 mM MgSO_4_ and 2.5 mM CaCl_2_ (LB/MC), unless otherwise stated. Serial dilution spot assays were performed to test phenotypes using overnight cultures grown in LB/MC that were first diluted to OD_600_ = 0.1, and then serial diluted from 10^0^ to 10^−5^; only the relevant dilutions are presented. Succinoglycan production and DOC sensitivity were assayed as described previously (16, 18). Temperature sensitivity was assayed on LB/MC/calcofluor/HEPES at 30°C and 40°C. Antibiotics were added to the media as appropriate as previously described (13, 15, 18, 20). GUS expression was assayed on LB/MC with 1 µg/mL X-Gluc (5-bromo-4-chloro-3-indolyl-beta-D-glucuronic acid cyclohexylammonium salt).

### Genetic techniques

All strains were created using either ΦN3 transduction or tri-parental mating as previously described (16). In order to move each divL and cckA suppressor into a clean CS6000 genetic background, a Tn*5* linked to either *divL* or *cckA* was isolated by the introduction of pLAFR::Tn*5* into HABsup9 and HABsup5, respectively, as previously described (33). Random Tn*5* mutants were pooled, and each pool was used to create a ΦN3 lysate for CS6000 transduction. Calcofluor-dim colonies were isolated, and >60% linkage to the calcofluor-dim phenotype was confirmed via transduction into CS6000.

The presence of each suppressor allele in CS6000 background was confirmed by PCR and DNA sequencing. These linked transposons were then used to transduce each *divL* and *cckA* allele into the wild-type Sm1021 genetic background to create single mutants.

The presence of each suppressor allele in the Sm1021 background was confirmed by PCR and DNA sequencing (Table 1).

**TABLE 1:** Strains, plasmids, and primers used in this study

Bacterial strain, plasmid, or primer	Genetic characteristics	Antibiotic resistance	Source or reference
*S. meliloti*			
Rm1021	Wild-type strain SU47	Sm	Ausubel
CS6000	Rm1021 Δ*cbrA*	Sm	(16)
CS7001	CS6000 *divL*^Q362P^	Sm	This study
HABsup1A	CS6000 *divL*^V602M^	Sm	This study
HABsup1B	CS6000 *divL*^F142S^	Sm	This study
HABsup2	CS6000 *divL*^L454I^	Sm	This study
HABsup3	CS6000 *divL*^F142S^	Sm	This study
HABsup5	CS6000 *cckA*^A373S^	Sm	This study
HABsup6	CS6000 *cckA*^A373S^	Sm	This study
HABsup7	CS6000 *cckA*^A373S^	Sm	This study
HABsup9	CS6000 *divL*^V787M^	Sm	This study
JP005	CS6000 *divL*^V602M^ Tn*5-divL*	Sm Nm	This study
JP006	CS6000 *divL*^L454I^ Tn*5-divL*	Sm Nm	This study
JP007	CS6000 *divL*^F142S^ Tn*5-divL*	Sm Nm	This study
JP008	CS6000 *divL*^V787M^ Tn*5-divL*	Sm Nm	This study
JP010	CS6000 *divL*^Q362P^ Tn*5-divL*	Sm Nm	This study
JP011	Rm1021 Tn*5-divL*	Sm Nm	This study
JP012	CS6000 Tn*5-divL*	Sm Nm	This study
JP013	Rm1021 *divL*^Q362P^ Tn*5-divL*	Sm Nm	This study
JP014	Rm1021 *divL*^F142S^ Tn*5-divL*	Sm Nm	This study
JP015	Rm1021 *divL*^L454I^ Tn*5-divL*	Sm Nm	This study
JP016	Rm1021 *divL*^V602M^ Tn*5-divL*	Sm Nm	This study
JP017	Rm1021 *divL*^V787M^ Tn*5-divL*	Sm Nm	This study
JP001	CS6000 *cckA*^A373S^ Tn*5-cckA*	Sm Nm	This study
JP002	Rm1021 Tn*5-cckA*	Sm Nm	This study
JP003	Rm1021 *cckA*^A373S^ Tn*5-cckA*	Sm Nm	This study
JP004	CS6000 Tn*5-cckA*	Sm Nm	This study
HAB2067	JP017 pBBR1-MCS-3	Sm Nm Tc	This study
HAB2069	JP017 pHAB3059	Sm Nm Tc	This study
HAB2031	Rm1021 pBBR1-MCS-3	Sm Tc	This study
HAB2028	Rm1021 pHAB3057	Sm Tc	This study
HAB2050	Rm1021 pHAB3059	Sm Tc	This study
HAB2022	CS6000 pHAB3059	Sm Tc	This study
HAB2035	CS6000 pBBR1-MCS-3	Sm Tc	This study
HAB2039	CS6000 pHAB3057	Sm Tc	This study
HAB2057	HABsup3 pBBR1-MCS-3	Sm Tc	This study
HAB2052	HABsup3 pHAB3059	Sm Tc	This study
HAB2045	CS7001 pHAB3059	Sm Tc	This study
HAB2058	CS7001 pBBR1-MCS-3	Sm Tc	This study
HAB2037	HABsup2 pBBR1-MCS-3	Sm Tc	This study
HAB2048	HABsup2 pHAB3059	Sm Tc	This study
HAB2043	HABsup1A pBBR1-MCS-3	Sm Tc	This study
HAB2054	HABsup1A pHAB3059	Sm Tc	This study
HAB2055	HABsup9 pHAB3059	Sm Tc	This study
HAB2059	HABsup9 pBBR1-MCS-3	Sm Tc	This study
HAB2056	HABsup6 pHAB3057	Sm Tc	This study
HAB2060	HABsup6 pBBR1-MCS-3	Sm Tc	This study
HAB2070	JP003 pBBR1-MCS-3	Sm Nm Tc	This study
HAB2074	JP003 pHAB3057	Sm Nm Tc	This study
MB672	Rm1021 pMB697	Sm Sp	(20)
HAB7010	Rm1021 pMB697	Sm Sp	This study
HAB7011	HABsup9 pMB697	Sm Sp	This study
HAB7012	HABsup6 pMB697	Sm Sp	This study
HAB7013	HABsup1A pMB697	Sm Sp	This study
HAB7024	CS6000 pMB697	Sm Sp	This study
HAB7025	HABsup3 pMB697	Sm Sp	This study
HAB7026	JP009 pMB697	Sm Sp	This study
HAB7027	HABsup2 pMB697	Sm Sp	This study
HAB8001	Rm1021 pJP1001	Sm Nm	This study
HAB8002	CS6000 pJP1001	Sm Nm	This study
HAB8003	CS7001 pJP1001	Sm Nm	This study
HAB8004	HABsup1A pJP1001	Sm Nm	This study
HAB8005	HABsup1B pJP1001	Sm Nm	This study
HAB8006	HABsup2 pJP1001	Sm Nm	This study
HAB8007	HABsup3 pJP1001	Sm Nm	This study
HAB8008	HABsup5 pJP1001	Sm Nm	This study
HAB8009	HABsup6 pJP1001	Sm Nm	This study
HAB8010	HABsup7 pJP1001	Sm Nm	This study
HAB8011	HABsup9 pJP1001	Sm Nm	This study
RH005	Rm1021 pK19ms::*dnaN*-mCherry	Sm Gm	This study
RH006	CS6000 pK19ms::*dnaN*-mCherry	Sm Gm	This study
*E. coli*			
MT616	MM294 pRK600	Cm	Finan
DH5α	*endA1 hsdR17 supE44 thi-1 recA1 gyrA relA1*Δ(*lacZYA-argG*)		BRL Corp.
BL21	(DE3) pLysS *ompT hsdSB* (*rB− mB−*) *gal dcm*	Cm	Invitrogen
Plasmids			
pBBR1-MCS-3	Low copy vector	Tc	(34)
pHAB3057	pBBR1-MCS-3::*cckA*^WT^	Tc	This study
pHAB3059	pBBR1-MCS-3::*divL*^WT^	Tc	This study
pSF-OXB20-HN2-V5-TEV	High Copy Expression vector, N-terminal V5	Kn	Oxford Genetics
pHAB4011	pSF-OXB20-HN2-V5-TEV:: *cckA*^ΔTM^	Kn	This study
pHAB4038	pSF-OXB20-HN2-V5-TEV:: *cckA*^ΔTM_A373S^	Kn	This study
pLAFR1	Low copy vector	Tc	(35)
pLAFR2070	*cbrA*^WT^ complementation vector	Tc	(18)
pVO155	Suicide vector with *uidA* cassette	Nm	(36)
pMB697	pVO345::*SMc00888-uidA*	Sp	(18)
pJB1001	pVO155::*ftsk2-uidA*	Nm	This study
pK19ms::*dnaN*-mCherry	pK19mobsacB with *dnaN*-mCherry translation fusion	Kn Gm	(12)
Primers	Sequence 5′–3′		
cat1	AACTCACCCAGGGATTGGCT		(16)
cat2	ACCAGACCGTTCAGCTGGATA		(16)
HAB0002	GCAGGCATTCAGTCGCT		This study
HAB0003	GGTACGCTTGATTCATCG		This study
HAB0004	ATGTCCTGACGGCCATC		This study
HAB0005	ACGACCTTCCGCATCCT		This study
HAB0007	AGCGACTGAATGCCTGC		This study
HAB0008	CGATGAATCAAGCGTACC		This study
HAB0009	GATGGCCGTCAGGACAT		This study
HAB0010	AGGATGCGGAAGGTCGT		This study
HAB0011	CAAACCGGAAACGGATTGGG		This study
HAB0012	TGAATGCGGGGTTCGGTTG		This study
HAB0015	GACTAGTCTGAACCAGGGTGAAGGTTGG		This study
HAB0016	GGGGTACCCCCGAGCTCTCGGAGGAACAGG		This study
HAB0017	CGAGCTCGCGGATACTGCTCATGAAAGG		This study
HAB0018	GACTAGTCAAGGGAGAGGAGGGCTTGTT		This study
HAB0021	GAATTCGATCGAGGTGATGCCGCAATCG		This study
HAB0022	GGATCCTCAGCTGTCGAGCATCTCGCG		This study
CTP050	ATAAGGATCCTGAACCAGGGTGAAGGTTGG		This study
CTP049	ATTACATATGGGCAAACAGACGGAGACAGA		This study
CTP019	AGGCCAGCTCGAAAAGGCGAT		This study
CTP020	AATCAGCGGCTGCAGTTCTACAAT		This study
CTP021	ATCCTCGACTACGCGGTCATT		This study
CTP022	TTCAACCGCTTCGAAAGCTAC		This study
CTP023	TGTCGGGTGCATCGGTTCGAA		This study
CTP024	GATGCCCCAGATCGCCCGGAA		This study
CTP025	AAACCTTGTCGCGGAAAGGCGTCT		This study
CTP026	TGTTCTTGTCGGCAATGAGAG		This study
HAB0023	GCAAAGAGGCGGGAGAGATT		This study
HAB0024	TCATCCACAGGAACCGCAAA		This study
HAB0025	CAGATGTGAACAAGTCGCCG		This study
HAB0026	TCCGCGGTCTGTTGATAAGG		This study
HAB0027	GATTTCTGGAGTTCTTGGGCG		This study
HAB0028	AGGCTTTTCACGTCGGGAG		This study
HAB0029	TTCTGGAGTTCTTGGGCGGT		This study
HAB0030	GCGACAAGGCTTTTCACGTC		This study
HAB0031	GGAGCGCTCGCAATTCATAC		This study
HAB0032	AGACCCTGCGGATCATCTCT		This study
HAB0033	GCAGGAGACGATGGATGGAA		This study
HAB0034	GAAACGCTGATCGCTCTCCA		This study
HAB0035	CGAGCTCCTTCGTGACTCTG		This study
HAB0036	AGAGAGGCGCAGACAATTCC		This study
HAB0037	CAGCCTCGCTTTCATGGAGA		This study
HAB0038	TCGGTAGAGGTGAACTTGCG		This study
HAB0039	GACGTGATCTGCAAGTCCCT		This study
HAB0040	CGGACCAATCTGCATCAGGA		This study
HAB0041	GAGCGGCACAAACAGGAATC		This study
HAB0042	CCTTGTTGGCCGGTTCAATG		This study
ftsK2-1	ACTAGTCCACTTCTGAAACGACAGC		This study
ftsK2-2	CTCGAGCGACGAACGCTGTATCAC		This study

### Molecular DNA techniques

The presence of Δ*cbrA* in suppressor mutants was confirmed by PCR using primers cat1 and cat 2 as previously described (16). Whole-genome sequencing was performed (Genewiz), and this identified *cckA* and *divL* mutations. The presence of these mutations was confirmed via PCR and sequencing (Eton Bioscience; CTP050/CTP049 and HAB0011**/**HAB0012, respectively). Native promoters for *divL* and *cckA* were predicted using Softberry BPROM (37), and complementation plasmids were designed to include these regions. *divL*^WT^ and *cckA* ^WT^ were amplified using HAB0015/HAB0016 and HAB0017/HAB0018, respectively. PCR products and pBBR1-MCS-3 were digested using SacI/SpeI or KpnI/SpeI for *cckA* and *divL*, respectively, creating pHAB3057 and pHAB3059. Primers HAB0021/HAB0022 were used to create CckA^ΔTM^ and CckA^ΔTM_A373S^ by deleting residues 1–75. PCR products and pSF-OXB20-NH2-V5-TEV were digested (EcoRI/BamHI) to create N-terminal V5-tagged protein fusions (pHAB4011 and pHAB4038). The upstream regulatory region of *ftsK2* was amplified using ftsK2-1 and ftsk2-2 primers. The resulting PCR fragment and pVO155 were digested using SpeI/XhoI and ligated together to create pJB1001.

### Symbiosis techniques

*M. sativa* symbiosis was assayed on BNM agar (28) as previously described (38). To isolate bacteria, nodules were removed and sterilized with 70% ethanol and then 20% bleach, rinsed several times, and placed in LB/MC supplemented with 0.3M glucose. Nodules were crushed, and serial dilutions of the bacterial suspension were grown on LB/MC/glucose with streptomycin, with one colony isolated from each nodule.

### Microscopy and flow cytometry

Cell morphology, DNA content, and DnaN-mCherry localization of cells in logarithmically growing batch culture were performed as previously described (16). Cell morphology was assayed as on a Zeiss Axioskop 2 Mot Plus microscope with a Hamamatsu Orca-ER camera using Openlab software. DNA content was quantified by FACS using cells stained with 0.5 µM SYTOX Green. One hundred thousand cells were acquired on a BD FACSAria II and subsequently analyzed using FlowJo.

### qPCR

qPCR analysis was performed on 25 ng of a whole-genome extraction from logarithmic batch cultures (OD_600_ = 0.6–0.8). Primers (10 mM) amplified the origin of replication (HAB0023/HAB0024) and terminus (HAB0027/HAB0028) of the chromosome along with *repABC* regions on the SymA (*repA2B2C2:* HAB0036/HAB0037, HAB0039/HAB0040, and HAB0041/HAB0042) and SymB (*repA1B1C1:* HAB0031/HAB0032, HAB0033/HAB0034, and HAB0035/HAB0036). SYBR-green was used to quantify PCR products and QuantStudio 3 Real-Time PCR System for data analysis. The 2^−ΔCT^ method was used for quantification and relative comparisons (39–41). The wild-type average value for each target was normalized to 1. For each mutant strain, the average value for each DNA target was normalized to wild type for the same target.

### Protein techniques

BL21 expressing CckA^ΔTM^ or CckA^ΔTM_A373S^ was grown at 30°C and 40°C. Cells were pelleted at 5,000 g for 10 mins at 4°C and resuspended in pre-chilled lysis buffer (42).

Samples were sonicated 3× for 30 s at 10% amplitude to generate a whole cell lysate, and then centrifuged at 16,000 × *g* for 25 min at 4°C to separate soluble from insoluble fractions. With Laemmli loading buffer added, samples were boiled for 10 min and separated by 10% SDS-PAGE. V5-tagged CckA^ΔTM^ or CckA^ΔTM_A373S^ protein in each sample was assessed by western blot analysis via transfer onto an Immobilon-FL Membrane. Membranes were incubated with monoclonal anti-V5 mouse antibody (1:1,000) in LI-COR Odyssey Blocking Buffer overnight at 4°C and subsequently treated with donkey anti-mouse IRDye 800CW antibody (1:20,000) for 2 hours at 4°C. Protein was visualized using the LI-COR detection system, and images were acquired with Image Studio 3.1. For enzymatic assays, V5-tagged CckA^ΔTM^ or CckA^ΔTM_A373S^ was immunoprecipitated from soluble fractions with anti-V5 agarose beads following the manufacturer’s supplied protocol (Sigma Aldrich). Enzymatic analysis of V5-tagged CckA^ΔTM^ and CckA^ΔTM_A373S^ was performed with ATP-γ-S following the manufacturer-supplied protocol (abcam) and as described (43). Samples were analyzed by western blot to quantify phosphorylated protein levels. Rabbit monoclonal thiophosphate ester (1:5,000) in Odyssey Blocking buffer at 4°C overnight, followed with goat anti-rabbit IRDye 800CW ab216773 (1:10,000). Following 2-hour incubation with 1 mM of ATP-γ-S at 30°C, 100 µM c-di-GMP was added to test for CckA phosphatase activity at 30°C. Samples were taken post-induction and probed for alkylated ATP-γ-S substrate via western blot using the LI-COR detection system, and images were acquired with Image Studio 3.1.

## SUPPLEMENTAL MATERIAL

Fig. S1 jb.00399-23-s0001.pdf Models for divL and cckA suppression of the cbrA mutant.

ASM does not own the copyrights to Supplemental Material that may be linked to, or accessed through, an article. The authors have granted ASM a non-exclusive, world-wide license to publish the Supplemental Material files. Please contact the corresponding author directly for reuse.
