# Supplementary material for: The Sinorhizobium meliloti nitrogen-fixing symbiosis requires CbrA-dependent regulation of a DivL and CckA phosphorelay
Source: J Bacteriol. 2024 Sep 24;206(10):e00399-23. doi: 10.1128/jb.00399-23 (PMC11500502; doi:10.1128/jb.00399-23)
Supplement: Fig. S1 — Models for divL and cckA suppression of the cbrA mutant. [file jb.00399-23-s0001.pdf]

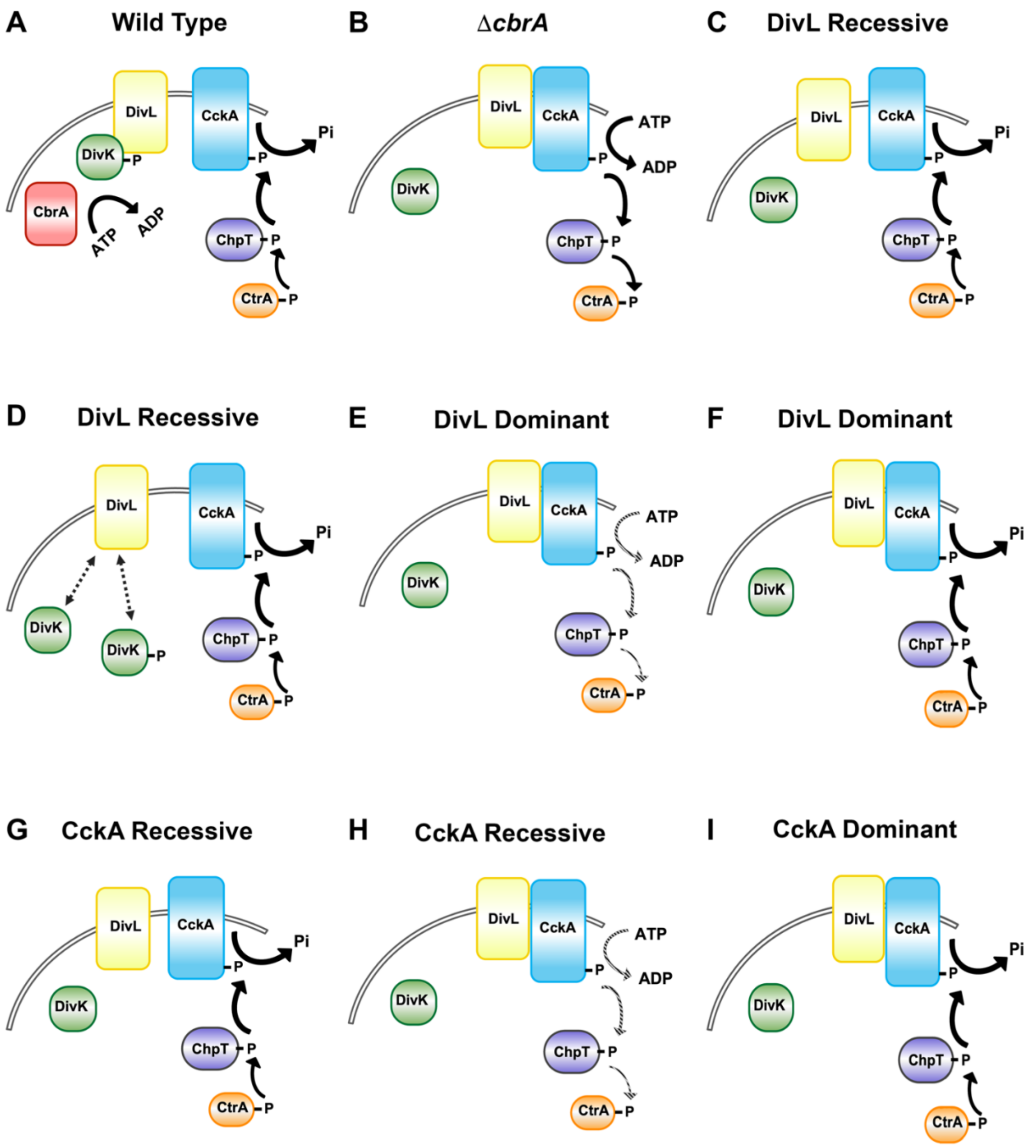


**FIG S1. Models of DivL and CckA predict mutant activities that would be either recessive or dominant to wild type.** In *C. crescentus*, DivL HisKA and Hatpase_c domains are required for DivK~P binding while PAS domains are crucial for DivK~P specificity over DivK (Fig. 3). In addition, DivL PAS domains appear to allosterically

regulate the CckA kinase/phosphatase switch (22, 24, 25). Thus, distinct DivL domains are responsible for sensing DivK phosphorylation status and communicating this to CckA. The *C. crescentus* CckA PAS-B domain is necessary for CckA localization to the new cell pole and efficient kinase activity in a manner dependent on DivL binding (Fig. 3) (22-26). This domain has also been implicated in c-di-GMP binding, which enhances CckA phosphatase activity (25). (A) In wild type, CbrA (along with DivJ, not shown), leads to phosphorylated DivK, titrating DivL away from CckA. This specific interaction between DivL/DivK~P allows CckA to act primarily as a phosphatase. (B) In Δ*cbrA*, the relative concentration of DivK~P decreases, allowing DivL to bind CckA and promote its kinase activity. (C) The hyperactive pathway resulting from Δ*cbrA* can be attenuated by recessive mutations in DivL that reduce CckA binding affinity. This would titrate some DivL mutant protein away from CckA and reduce its kinase activity. This mechanism is expected to be recessive since $DivL^{WT}$ would be able to bind CckA and reestablish high CtrA activity. (D) Similarly, Δ*cbrA* can be attenuated by recessive mutations in DivL that reduce its ability to differentiate DivK from DivK~P. (E) The hyperactive pathway resulting from Δ*cbrA* can be attenuated by dominant mutations in DivL that can bind CckA but are unable to switch its partner from phosphatase to kinase activity, or (F) enhances CckA phosphatase activity upon binding. These mechanisms of suppression would display semi-dominant to dominant phenotypes with the relative strength depending on DivL mutant protein affinity for CckA and its ability to compete with $DivL^{WT}$ for binding. (G) The hyperactive pathway resulting from Δ*cbrA* can be attenuated by recessive mutations in CckA that reduce DivL binding affinity which would favor phosphatase activity and thereby decrease CtrA activity. This mechanism for

suppressing Δ*cbrA* would be expected to be recessive since $CckA^{WT}$ would be able to bind $DivL^{WT}$ and restore high CtrA activity. (H) Similarly, Δ*cbrA* can be attenuated by recessive mutations in CckA that significantly decrease its inherent kinase activity even when bound to DivL. (I) The hyperactive pathway resulting from Δ*cbrA* can be attenuated by mutations in CckA that enhance inherent phosphatase activity even when bound to DivL. These CckA alleles may either have a conformational defect or increased binding affinity for c-di-GMP and would be semi-dominant or dominant with the outcome depending on the intracellular ratio of $CckA^{A373S}$ to $CckA^{WT}$.
